# CellCover defines marker gene panels capturing developmental progression in neocortical neural stem cell identity

**DOI:** 10.7554/eLife.107531

**Published:** 2026-07-28

**Authors:** Lanlan Ji, An Wang, Shreyash Sonthalia, Seungmae Seo, Daniel Q Naiman, Laurent Younes, Carlo Colantuoni, Donald Geman

**Affiliations:** 1 Department of Applied Mathematics and Statistics, Johns Hopkins University Baltimore United States; 2 Departments of Neurology and Neuroscience, Johns Hopkins University Baltimore United States; 3 Department of Natural Sciences, University of Maryland Eastern Shore Princess Anne United States; 4 Center for Imaging Science, Johns Hopkins University Baltimore United States; 5 Institute for Genome Sciences, University of Maryland School of Medicine Baltimore United States; Seoul National University Republic of Korea; Seoul National University Republic of Korea

**Keywords:** human, primate, mouse, single-cell RNA-seq, marker genes, neocortex, Human

## Abstract

Defining cell classes is central to the analysis of growing single-cell RNA sequencing (scRNA-seq) atlases. Marker genes are most often identified by differential expression (DE) methods that assess genes one at a time, ignoring the redundancy and complementarity revealed when genes are considered jointly. Working with binarized expression data, we instead seek discriminating *panels* of genes that together are specific to a cell type, framing marker-panel selection as a variant of the minimal set-covering problem in combinatorial optimization. This formulation efficiently searches the vast space of candidate panels, exploits the large cell numbers typical of scRNA-seq, and is robust to zero-inflation. Using blood and brain data, we show that our method, CellCover, reduces gene redundancy and captures cell-class-specific signals distinct from those found by DE. Transfer-learning experiments across mouse, primate, and human data demonstrate that CellCover identifies conserved cell classes in neocortical neurogenesis and tracks developmental progression in progenitors and neurons. Examining outer radial glia markers across mammals, we find that transcriptomic elements of this key cell type likely arose in rodent gliogenic precursors before the full program emerged in the primate lineage.

## Introduction

Single-cell RNA sequencing (scRNA-seq) technologies provide measurements of RNA molecules in many thousands of individual cells, yielding a rich source of information for determining attributes of cell populations, such as cell types and the variation in gene expression from cell to cell, which are not available from bulk RNA sequencing data (Angerer et al., 2017; Grün and van Oudenaarden, 2015; Regev et al., 2017; Svensson et al., 2018; Wang and Navin, 2015). After standard preprocessing and normalization, a core challenge in the analysis of scRNA-seq data is to annotate the cells in a given dataset with a label indicating cell class, type, or state. This usually involves multiple phases of processing, notably dimension reduction, clustering, and finding ‘marker genes’ for the labels of interest. Markers are usually selected by first ranking individual genes based on a statistical test of differential expression (DE) across cell type labels and then selecting a panel of top-ranking genes based on manual curation, estimates of predictive capacity, a priori information (e.g. cell-surface proteins), or simply panel size (Delaney et al., 2019; Luecken and Theis, 2019; Shekhar et al., 2016; The Tabula Muris Consortium et al., 2018; Grønbech et al., 2020; Satija et al., 2015; Wang and Navin, 2015; Yuan et al., 2017). Importantly, the construction of such marker panels incorporates data from only one gene at a time, that is the panel is assembled ‘gene by gene’, and does not take into account possible gene combinations that could better distinguish cell classes.

Complications arise because scRNA-seq data are high dimensional (Becht et al., 2018), highly heterogeneous, generally noisier than bulk RNA-seq, and stochastically zero-inflated. Complex pipelines with many choices require manual intervention (Delaney et al., 2019; Dumitrascu et al., 2021) and raise issues of bias and reproducibility (Abdelaal et al., 2019; Stegle et al., 2015). In order for downstream analyses to be computationally feasible, restricting dimension, such as using small panels of marker genes, is necessary. Analysis of scRNA-seq data is generally done at the univariate (single gene) level, even though the relevant biology is often decidedly multivariate. In particular, probability distributions, when proposed, are for the expression of individual genes (i.e. marginal distributions) not for gene panels (i.e. higher-order marginals). These analyses do not take into account how marker genes may cooperate to define cell types, or possible heterogeneity within cell types.

To avoid these limitations, we formulate marker gene selection as a variation of the well-known ‘minimal set-covering problem’ in combinatorial optimization. In our case, the ‘covering’ elements are genes, and the objects to be covered are the cells in a particular class. A cell is covered at depth $\boldsymbol{d}$ by a gene panel if at least $\boldsymbol{d}$ genes in this panel are expressed (raw count ≥ 1) in the cell (Appendix 1). A set of genes is then a depth $\boldsymbol{d}$ marker panel for a cell type if nearly all cells of that type are covered at depth $\boldsymbol{d}$. We define the covering rate as $1-\boldsymbol{\alpha}$, where **α** represents the fraction of uncovered cells, that is the false negative rate. This covering rate can be adjusted to balance specificity and sensitivity of the marker genes. Prior to selection of the gene panel, a weight is calculated for each gene, defined as its expression outside the target class divided by its expression within that class. Lower weights, therefore, correspond to greater discriminative power. This weight calculation can be carried out as one class versus all other cells (as used in this report), or as one class versus its closest neighboring class, and can be based on either binary or count-based expression. The optimal marker panel is then selected from genes with higher expression within the cell type of interest (weight <1), determined by minimizing the sum of weights in the gene panel while ensuring that the panel achieves the desired covering rate. We formulate this search as a constrained optimization problem constructed as an integer linear program (Cormen et al., 2022; Ke et al., 2021; see Computing marker sets in Methods). This approach enables the identification of compact sets of marker genes that reliably distinguish cell populations. Additionally, gene panels can be transferred across studies to explore shared cell-type-specific expression patterns by examining covering depth of the panels in new cells and covering rates in new cell classes.

The minimal covering approach in CellCover is designed specifically to leverage the high number of cells routinely observed in scRNA-seq data in order to be robust to the stochastic zero-inflation known to be pervasive in this data modality: Because the covering algorithm optimizes a set of genes rather than individual markers and different true marker genes will have drop-outs in different cells, it follows that if many cells of a class are sequenced, zero-inflation can be overcome (while also mitigating the impact of higher noise at low expression levels). In contrast, standard methods, based on selecting genes for which a DE statistic is extreme, cannot borrow strength across genes in a marker panel when selected gene-by-gene. CellCover searches for small panels of covering marker genes that precisely define cell classes together as a set, and are also expandable, that is can be readily linked to additional related genes for the interrogation of cell-type-specific function. In addition, CellCover output can be used in gene set over-representation analysis and the deeper exploration of heterogeneity within individual cell classes.

To demonstrate CellCover’s utility in identifying cell-type-specific markers, we benchmark CellCover against leading marker selection methods using single-cell RNA-seq datasets from human blood. We show that CellCover effectively reduces marker redundancy and outperforms most methods in predicting cell types. In a series of in silico experiments, we use CellCover to explore cell classes and their developmental progression across neurogenesis in the excitatory neuronal lineage of the mammalian neocortex. Through cross-dataset marker transfer experiments, we validate the robustness and transferability of CellCover markers across various sequencing protocols and biological contexts, highlighting its capacity to consistently map cell-type-specific gene expression from a reference dataset to cells or samples in new target datasets.

## Results

### CellCover on blood scRNA-seq data: benchmark and transfer

#### Benchmarking cell type definition

**Figure 1.**
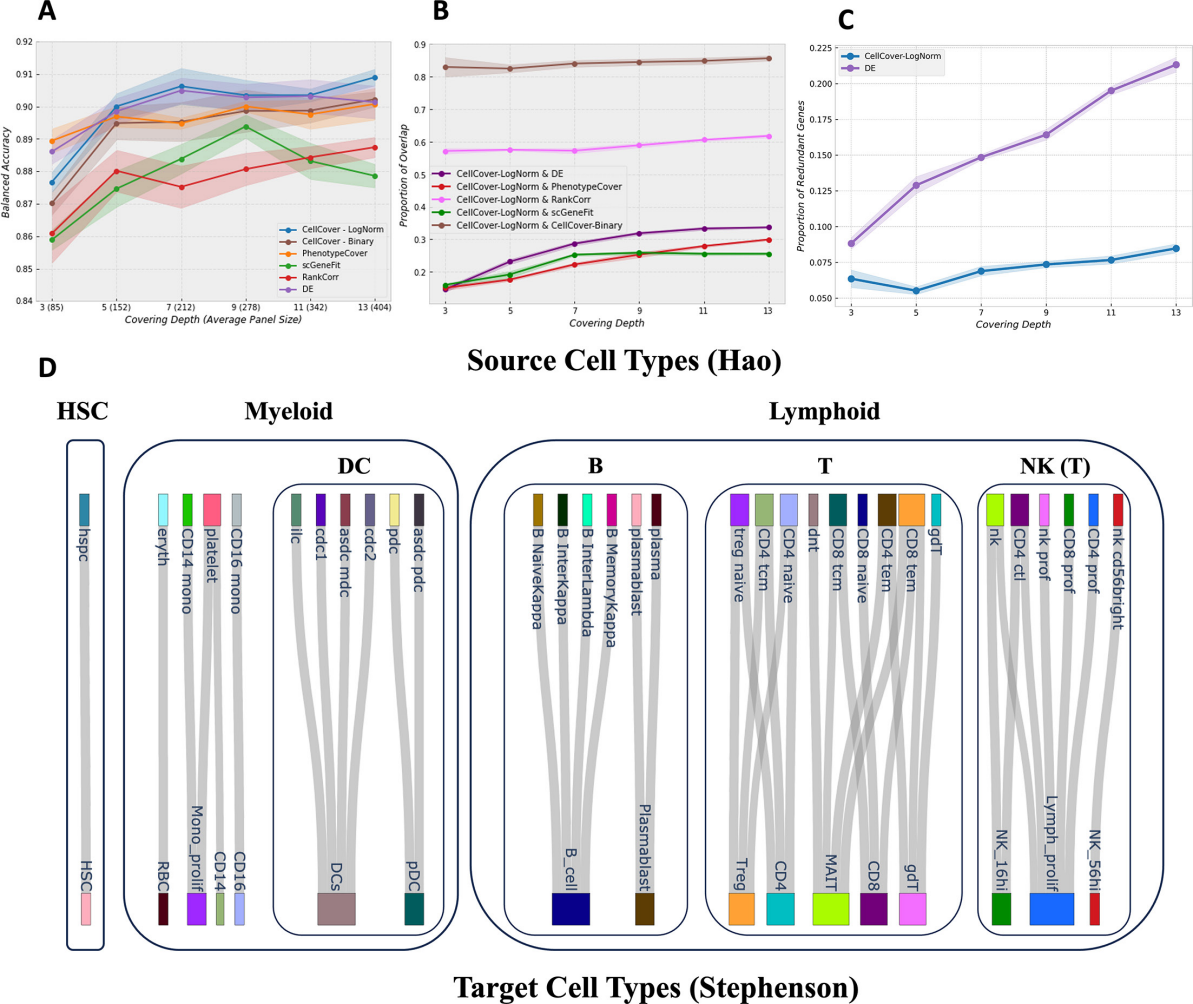
Benchmarking performance of CellCover and competing methods on the CBMC dataset and blood cell type mapping. (**A**) Balanced accuracy versus the size of the marker panel for all methods, with SD across random seeds shown as shaded areas. CellCover implemented with weights calculated from log normalized or binary data are both shown. (**B**) Proportion of intersection between each method’s global marker panel and the CellCover (log-normalized) reference panel. (**C**) Proportion of redundant marker genes selected for multiple cell classes versus panel size for CellCover and DE, also including SD across random seeds. (**D**) Sankey diagram of the mapping from source blood cell types (Hao et al., 2021) to target blood cell types (Stephenson et al., 2021). Original authors’ cell type labels are used: HSC = hematopoietic stem cells, DC = dendritic cells, B=B cells, T=T cells, NK = natural killer cells, eryth +RBC = red blood cells, mono = monocytes, ilc = innate lymphoid cells, prof +prolif = proliferating.

We compare CellCover with leading marker gene selection algorithms – Wilcoxon rank sum-based DE (Wolf et al., 2018), RankCorr (Vargo and Gilbert, 2020), scGeneFit (Dumitrascu et al., 2021), and PhenotypeCover (Hasanaj et al., 2022) – to evaluate their effectiveness in identifying cell state resolving markers. This benchmark analysis uses the CBMC CITE-Seq dataset (Stoeckius et al., 2017), which provides a rich source of immune cell information from cord blood mononuclear cells (CBMCs) through multimodal single-cell analysis. To systematically assess the different methods, we employ a fivefold cross-validation approach to first extract marker genes from the training data, using only the RNA expression, and then test cell type label recovery in test data using these markers in a support vector machine (SVM). To generate marker gene panels, we implemented CellCover at various depths of coverage with a cover rate of 90% ($\boldsymbol{\alpha}=0.1$), using both log-normalized and binary expression data to define gene weights (see Gene weight computation based on binary and log-normalized expression profile in Methods). For DE, we selected the top genes for each cell type, matching the exact number of markers in the CellCover panel for that cell type. Unlike DE and CellCover, the RankCorr, scGeneFit, and PhenotypeCover methods do not generate cell-type-specific marker panels but rather global panels that distinguish all cell types. Therefore, at each covering depth, we select an equivalent number of markers for RankCorr, scGeneFit, and PhenotypeCover to match the global CellCover panel size (i.e. the total number of unique marker genes used for all cell classes combined). After selecting marker gene panels for all methods, we trained an SVM (see Support vector machine in Methods) on the training data using only the marker genes identified by each method, and then report the average balanced accuracy (see Balanced accuracy in Methods) on the test data restricted to these markers. The results in Figure 1A show that CellCover, along with DE methods, consistently achieves the highest balanced accuracy at different marker panel sizes. Importantly, while CellCover and DE methods achieve similar levels of performance in cell type definition, they do so by utilizing distinct sets of genes, selecting only 20–30% of the same genes in their marker panels (Figure 1B; see Intersection of global marker panels in Methods). This indicates that CellCover and DE methods leverage quite different elements of gene expression to distinguish cell types.

**Figure 1—figure supplement 1.**
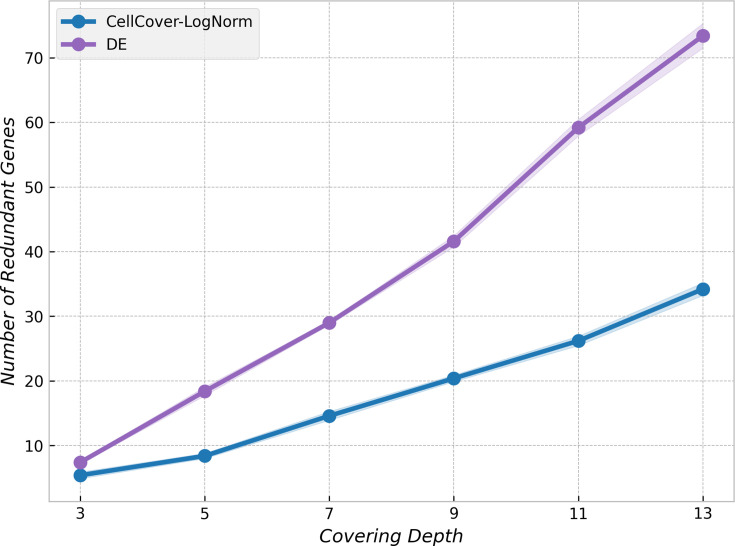
Number of redundant marker genes versus panel size for CellCover and DE.

**Figure 1—figure supplement 2.**
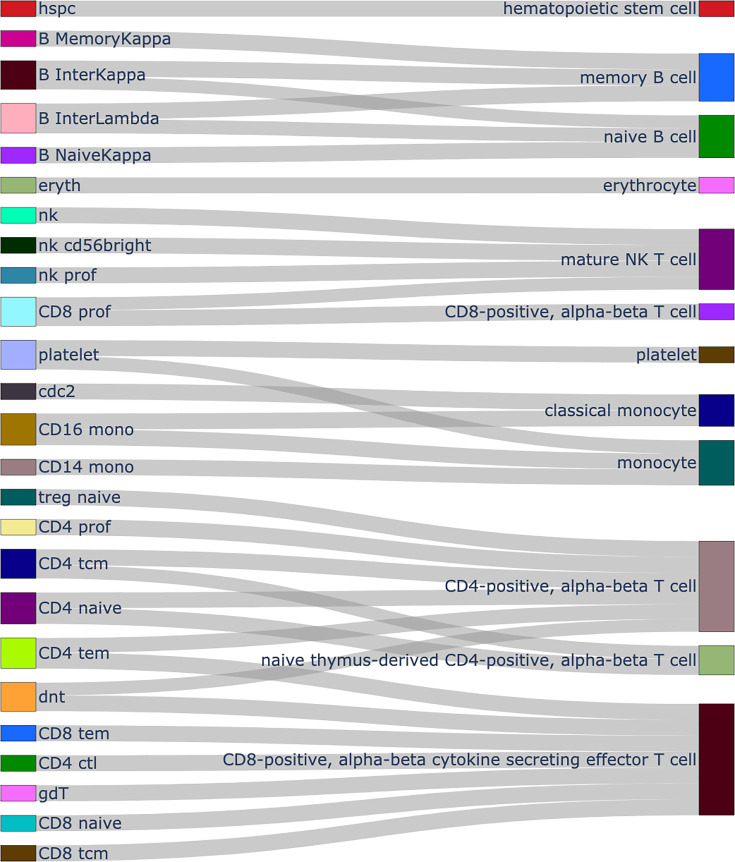
Sankey diagram depicting cell type mapping from the Hao PBMC CITE-seq dataset to the Tabula Sapiens blood dataset (Smart-seq protocol).

**Figure 1—figure supplement 3.**
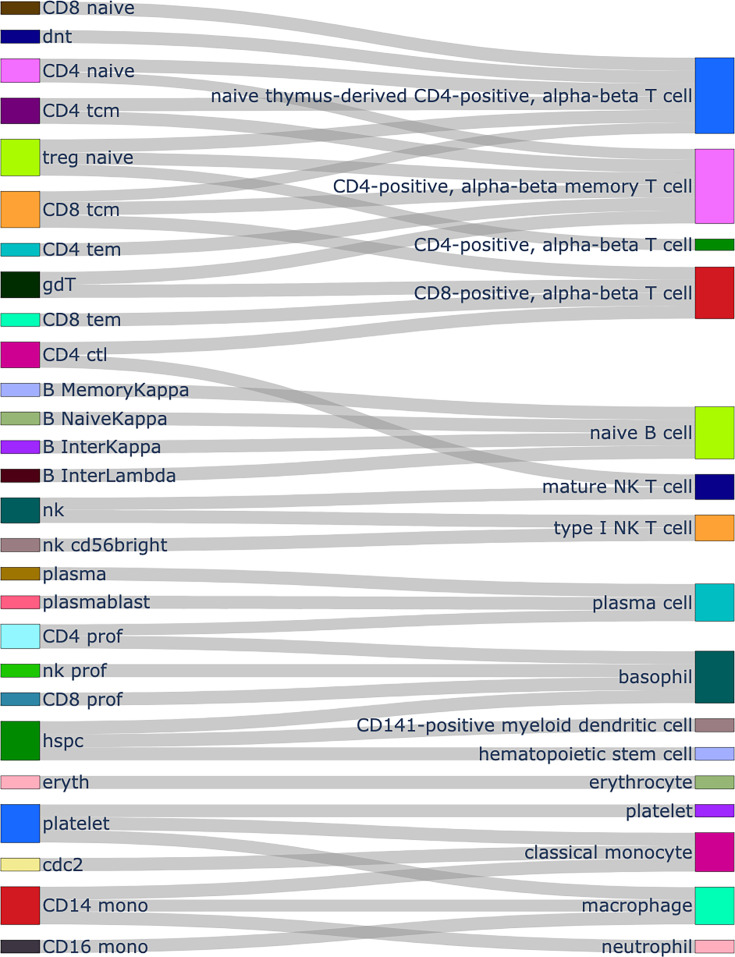
Sankey diagram depicting cell type mapping from the Hao PBMC CITE-seq dataset to the Tabula Sapiens blood dataset (10x5’ protocol).

**Figure 1—figure supplement 4.**
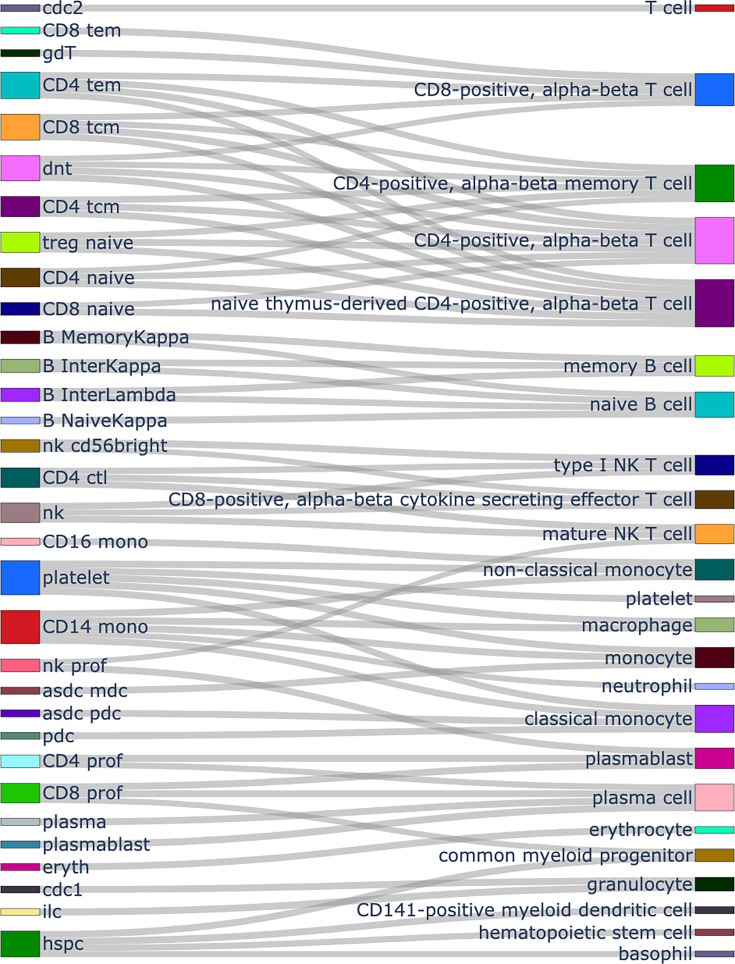
Sankey diagram depicting cell type mapping from the Hao PBMC CITE-seq dataset to the Tabula Sapiens blood dataset (10x3’ protocol).

In addition to optimal cell class label recovery, it is desirable that marker gene panels do not contain the same genes for different cell classes. Because DE methods choose marker genes one at a time from a ranked list of statistics for each cell class, they often select the same gene for more than one cell class. This is especially true if some cell classes are more similar to one another than to other classes, which is a common scenario in many biological contexts. As evidenced by Figure 1—figure supplement 1, CellCover selects fewer redundant marker genes compared to DE at all covering depths, indicating that its marker panels capture more unique, non-overlapping signals across cell types. Figure 1C provides another view of this, showing that the proportion of redundant CellCover markers remains stable around 7.5%, while the proportion of redundant DE markers steadily increases with the marker panel size, surpassing 20% in larger panels (see Within marker panel redundancy in Methods). This greatly reduced redundancy demonstrates that CellCover generates concise marker panels in which genes provide distinct and complementary information.

#### Cell type mapping across datasets through marker transfer

To demonstrate that CellCover effectively captures robust cell-type-specific information that is transferable across diverse single-cell RNA-seq datasets, we performed cross-dataset marker transfer experiments between datasets derived from human blood. Using the Hao PBMC CITE-seq dataset (Hao et al., 2021) as a source dataset with marker panels generated at depth 5 and a covering rate of 98%, we evaluated the ability of these CellCover marker panels to correctly identify cell types in four new target datasets: The Tabula Sapiens blood data (Jones et al., 2022) sequenced with three protocols (smart-seq2, 10x3’, and 10x5’) and the Stephenson COVID-19 PBMC dataset (Stephenson et al., 2021), restricted to cells from healthy controls (Appendix 2; NeMO: Individual genes in blood and NeMO: Blood 34 CellCover Panels).

The Sankey diagrams in Figure 1, Figure 1—figure supplements 2–4 (see Cell type mapping across datasets in Methods) demonstrate that marker gene panels identified by CellCover accurately map cell types from the source dataset to cell types of the same hematopoietic lineage in the target datasets (despite divergent cell type labeling of the original studies). This can be seen in Figure 1D where cell types of common lineage origin are very rarely incorrectly mapped, that is cell type mappings stay within hematopoietic lineages noted in the figure. The one exception to this is that a small proportion of the DCs in the target dataset were mapped to innate lymphoid cells (ilc), a cell type that is not well-defined and extremely rare in the circulating blood (Vivier et al., 2018). This result indicates the robustness of CellCover marker gene panels in mapping cell-type-specific signals across different sequencing technologies and experiments.

### scRNA-seq datasets in neocortical neurogenesis

We illustrate the use of CellCover in the context of dorsal telencephalic neurogenesis during mid-gestation, when the majority of excitatory neurons of the neocortex are produced. The mature neocortex in mammals is made up of six layers of post-mitotic neurons. Neurons of each new layer are produced in succession, with neurons of the new layer migrating past previously created layers, leading to an ‘inside-out’ arrangement of neurons by birthdate (Angevine and Sidman, 1961), with deep layers (VI-V) being born first and superficial layers (II-IV) born last. Neurons are generated sequentially from radial glia (RG), the neural stem cells of the telencephalon, and then intermediate progenitor cells (IPCs) through both symmetric and asymmetric cell division (Lancaster and Knoblich, 2012). The number and diversity of neural stem and progenitor cells in the cortex have been greatly expanded in the evolution of the primate lineage. In particular, the appearance of the outer subventricular zone (OSVZ) in primates appears to underlie the development of the supragranular layers of the cortex (layers II and III), which contribute to higher cognition through extensive cortico-cortico connections (Betizeau et al., 2013). Notably, this includes greater proliferative capacity of neural stem cells and the expansion of outer radial glia (oRG, or basal radial glia, bRG), which are rare in rodents but numerous in primates and especially in humans (Wang et al., 2011; Hansen et al., 2010). As each cortical layer is generated, newborn neurons arise contemporaneously from progenitor cells in the germinal zones (GZ) and migrate radially along processes of the RG, outward toward the surface of the telencephalon, to the cortical plate (CP), coming to rest together at their characteristic position in the developing cortex (Rakic et al., 2009). Neurons arising at the same time and place, and therefore from precursors with common molecular features, share unique morphological and functional characteristics. We are interested both in the general process by which neural precursor cells yield post-mitotic neurons, and in the more nuanced dynamics by which specific properties are imparted to neurons which are born together and which come to rest in the same cellular microenvironment, that is cortical region and layer.

How the molecular dynamics of neural progenitor identity across this progression impact the fate and function of the neurons being produced is not fully understood. To explore both the principal cell identities and the developmental progression within cell types, we first focus on scRNA-seq data from Telley et al., 2019 containing cells extracted during neocortical neurogenesis in the mouse, and for which highly precise developmental metadata are available. This dataset contains expression data from cells produced across 4 days of mid-gestational embryonic development during the peak of excitatory neocortical neurogenesis (E12-15). Using a technique to specifically label ventricular RG cells during their final neurogenic cell division, cells born on each of the four embryonic days were sampled at 1 hr, 24 hr, and 96 hr after this terminal division. This yielded a two-dimensional temporal indexing of the data based on the embryonic age of the animals and the precise time since the final division of the individual cells sequenced. We define CellCover marker gene panels in the principal cell types involved in neocortical neurogenesis as they progress through development and then validate their ability to capture conserved cell-type-specific signals in additional scRNA-seq data from developing mouse (La Manno et al., 2021; Di Bella et al., 2021), primate (Bakken et al., 2016; Micali et al., 2023), and human (Polioudakis et al., 2019; Jaffe et al., 2018) cortical tissue. In order to focus on cell states and transitions in excitatory neurogenesis, for all the studies we use in this report, we attempt to analyze only cells of the excitatory neurogenic lineage, omitting inhibitory neurons and later astroglial and oligodendroglial cells, as well as non-neural cells including microglia and endothelial cells. Individual gene expression data in all the public studies we employ here can be explored without coding expertise at: NeMO: Individual genes in cortex.

### CellCover marker gene panels in mouse neocortical development #1: Cell types

The precise cell birth dating that is coupled to scRNA-seq data in the Telley study (Telley et al., 2019) provides a unique opportunity to interrogate cell type transitions during neocortical neurogenesis. To take advantage of this experimental design, rather than using data-driven clusters or biologist-annotated cell labels, we employed the empirical timing data for individual cells to define cell classes. At 1 hr after final division, it is likely that the transcriptome of RGs is still representative of the progenitor state, while at 96 hr, we expect cells to have transitioned to a postmitotic neuronal state. Cells at 24 hr after final division represent transitional states between the RGs and postmitotic neurons, likely spanning intermediate progenitor, neuroblast, and nascent neuronal states. Using our novel CellCover approach, we generated marker gene panels defining cell states at 1, 24, and 96 hr after the terminal division of ventricular RGs (regardless of embryonic day).

**Figure 2.**
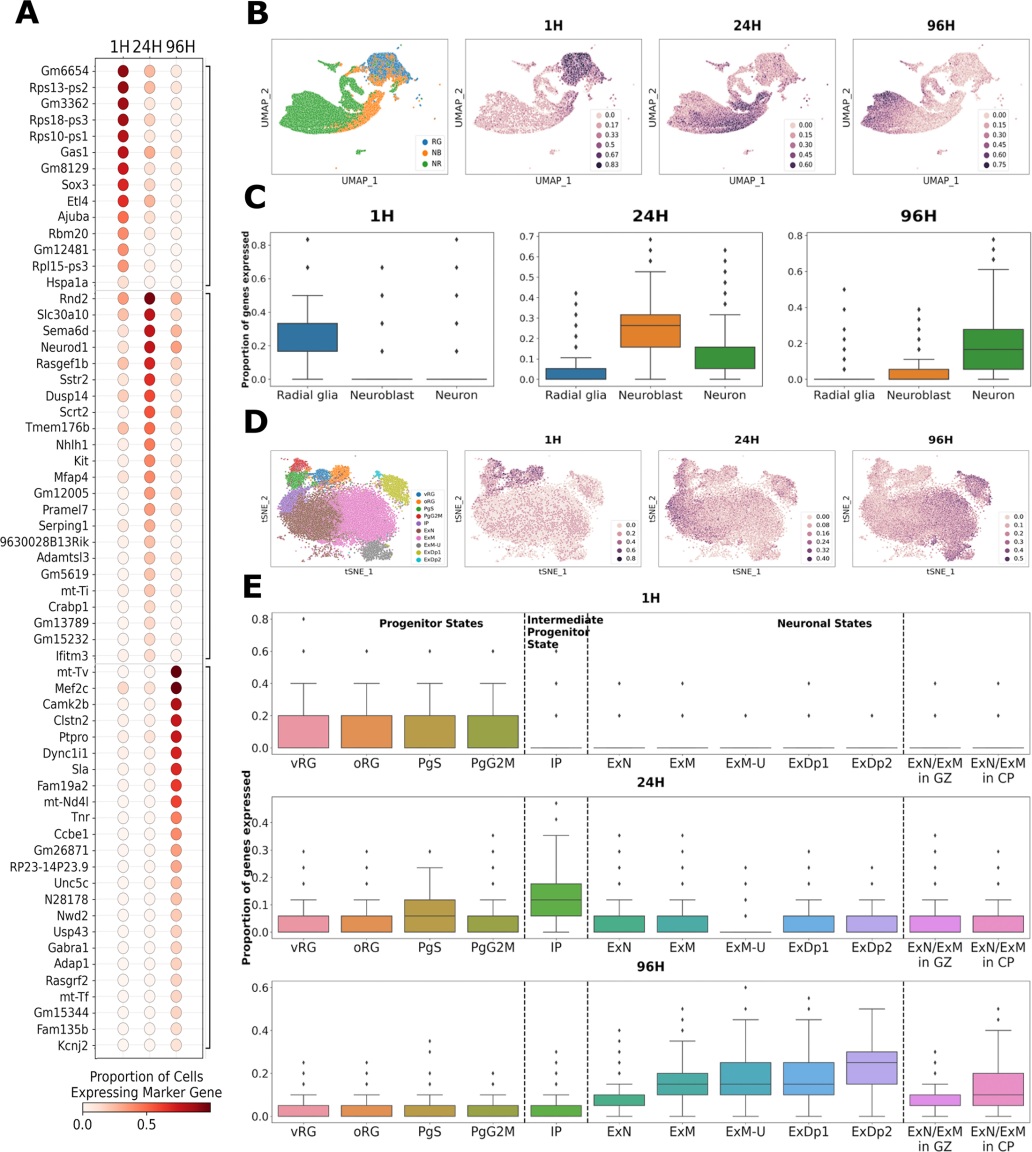
CellCover marker gene panels in the developing mammalian neocortex. (**A**) Dot plot of empirical conditional expression probabilities of CellCover marker panels of each cell age. The marker genes of each cell age are grouped along the horizontal axis and sorted by expression frequency in the cell class of interest. The color of the dots represents the expression probability of markers conditioned on time since a cell’s terminal division, i.e., the proportion of cells within a class expressing the marker gene. For this analysis, cells of each time point were pooled across embryonic ages (E12–15). The panel is obtained using the CellCover with $\boldsymbol{\alpha}=0.02$ and $\boldsymbol{d}=5$. RG = radial glia, NB = neuroblast, NR = neuron. (**B**) Transfer of marker gene panels from the Telley data (Telley et al., 2019) to a second mouse neocortex dataset shows consistent identification of the primary cell types in mouse neurogenesis. Left: UMAP representations of cells from the dorsal forebrain excitatory lineage in the La Manno atlas of mouse brain development (La Manno et al., 2021), colored by cell labels assigned by the original authors. This is followed by the same UMAPs, now showing the proportion of gene panels derived from the Telley dataset, labeled 1 H, 24 H, and 96 H, expressed at non-zero levels in each individual cell. These last three plots illustrate the transfer of marker gene panels derived from the Telley data to the La Manno data. (**C**) Box plots of these same proportions broken down by cell-type labels provided by the original authors. (**D**) Transfer of marker gene panels from the Telley data in mouse to data in the developing human neocortex from Polioudakis et al., 2019, shows the identification of conserved cell types in neurogenesis across mammalian species. Left: tSNE map of cells from the dorsal forebrain excitatory lineage in the Polioudakis data. This is followed by the same tSNE maps, now showing the transfer of marker gene panels derived from progenitors labeled 1, 24, and 96 hr after terminal cell division in the Telley data. The map is colored by the proportion of each gene panel that is expressed at non-zero levels in each individual cell of the human Polioudakis dataset. Original author cell labels are used: RG = radial glia, v=ventricular, o=outer, Pg = cycling progenitor, S=in S phase, G2M=in G2M phase, IP = intermediate progenitor, Ex = excitatory neuron, N=new migrating, M=maturing, Dp = deep layer, U=upper layer. (**E**) Box plots of these same proportions broken down by cell type labels and microdissection information provided by the original authors. The final two boxes indicate expression in neuronal subtypes segregated by physical location: germinal zone (GZ) or cortical plate (CP) microdissection.

Figure 2A depicts the gene panels derived from cells at each of the three times sampled, shown as the proportion of cells at each time that express each of the marker genes at non-zero levels. The original authors noted elevated heterogeneity in the 24 H cell class, which can be seen as more frequent expression of 24 H cell markers in the other time classes. This is consistent with the 24 H cells having their own transcriptomic characteristics, while also harboring elements of both the preceding progenitor state and the subsequent neuronal state. The expression of these marker gene panels can be viewed across the public datasets we use in this report at: NeMO: Telley 3 CellCover Panels.

**Figure 2—figure supplement 1.**
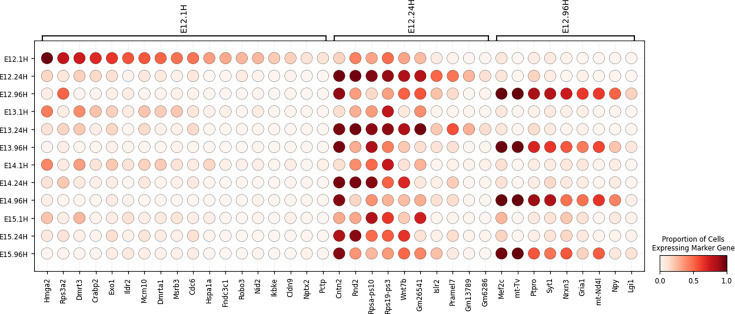
Dot plot of CellCover marker gene panels for three cell ages (1, 24, and 96 hr) at embryonic day E12 in mouse neocortical development. The marker genes of each cell age at its corresponding embryonic day are grouped along the horizontal axis and sorted by expression frequency in the cell class of interest. The color of the dots represents the expression probability of covering markers conditioned on time since a cell’s terminal division. The panel is obtained using the CellCover with $\alpha=0.02$ and $d=5$.

**Figure 2—figure supplement 2.**
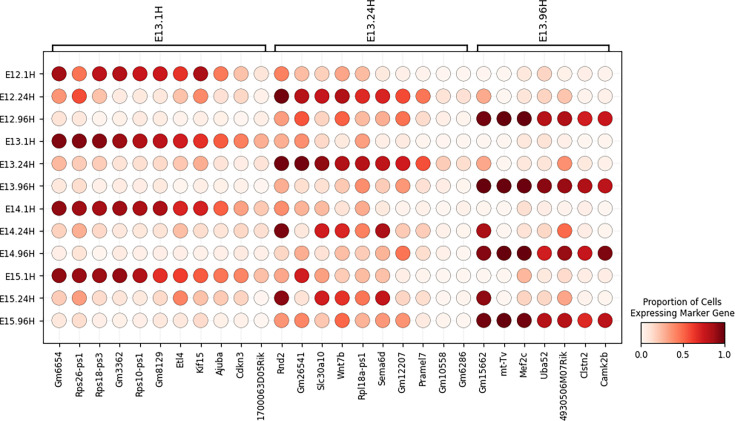
Dot plot of CellCover marker gene panels for three cell ages (1, 24, and 96 hr) at embryonic day E13 in mouse neocortical development. The marker genes of each cell age at its corresponding embryonic day are grouped along the horizontal axis and sorted by expression frequency in the cell class of interest. The color of the dots represents the expression probability of covering markers conditioned on time since a cell’s terminal division. The panel is obtained using the CellCover with $\alpha=0.02$ and $d=5$.

**Figure 2—figure supplement 3.**
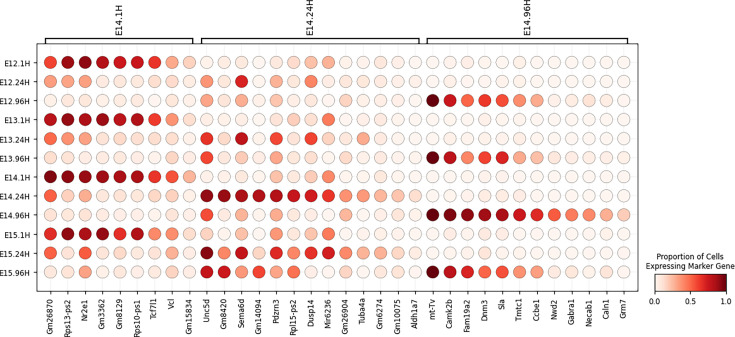
Dot plot of CellCover marker gene panels for three cell ages (1, 24, and 96 hr) at embryonic day E14 in mouse neocortical development. The marker genes of each cell age at its corresponding embryonic day are grouped along the horizontal axis and sorted by expression frequency in the cell class of interest. The color of the dots represents the expression probability of covering markers conditioned on time since a cell’s terminal division. The panel is obtained using the CellCover with $\alpha=0.02$ and $d=5$.

**Figure 2—figure supplement 4.**
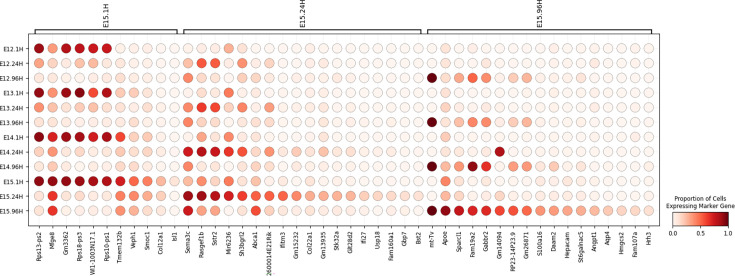
Dot plot of CellCover marker gene panels for three cell ages (1, 24, and 96 hr) at embryonic day E15 in mouse neocortical development. The marker genes of each cell age at its corresponding embryonic day are grouped along the horizontal axis and sorted by expression frequency in the cell class of interest. The color of the dots represents the expression probability of covering markers conditioned on time since a cell’s terminal division. The panel is obtained using the CellCover with $\alpha=0.02$ and $d=5$.

These marker gene panels contain canonical markers of neural progenitors, intermediate progenitors/neuroblasts, and neurons, including *Sox3*, *Neurod1*, and *Mef2c* in 1 H, 24 H, and 96 H cells, respectively. The panels also contain many lesser-known genes (*Gm* …), pseudogenes (…-*ps*), and mitochondrial-encoded genes (*mt*-…) which are expressed at much lower levels, but together precisely distinguish these neurogenic cell classes. Mitochondrial transcripts, and tRNAs in particular, are often disregarded as uninformative ‘housekeeping’ genes or even indications of low-quality RNA samples. These are dangerous assumptions, especially when exploring neurogenesis where the mitochondrial content of cells changes dramatically as cells transition from stem and progenitor states to post-mitotic neurons. The expression of mt-Tv, for example, is highly specific to 96 H cells (Figure 2A), and while often disregarded as a housekeeping tRNA, it also forms a structural component of the mitochondrial ribosome (Brown et al., 2014) and is transcribed in higher abundance as energy demands of nascent neurons increase. This suggests that many genes not often selected by conventional marker gene finding methods (and often intentionally filtered out by biologists) hold significant cell-type-specific information. To assess this possibility and the utility of CellCover marker gene panels in general, we next examine the ability of these panels to interrogate cell-type-specific signals in additional neocortical datasets.

### Transfer of CellCover marker gene panels #1: conserved cell types in neocortical neurogenesis

To assess the ability of CellCover marker gene panels to capture consistent cell-type-specific signals in murine neurogenesis, we explored additional in vivo data from the dorsal mouse telencephalon. We examined the expression of CellCover panels derived from the Telley mouse dataset in a recent atlas of the developing mouse brain (La Manno et al., 2021; Figure 2B and C). For both these mouse scRNA-seq datasets, the precise gestational age of the mouse pup of origin is known for all cells collected. However, while there are ground truth labels of the time from ventricular progenitor terminal cell division in the Telley data, in the atlas we must rely on cell type annotation provided by the researchers, which include RG, neuroblast, and neuron classes sequentially along the neurogenic trajectory. As expected, marker genes derived from progenitors labeled one hour after their terminal cell division in the Telley data, that is while still in the progenitor state, were most frequently expressed in the progenitors (i.e. RG cells) of the mouse dorsal forebrain atlas. Correspondingly, markers of 24 H cells were most frequently expressed in neuroblasts (and some neurons, as noted by original authors), and markers of 96 H cells in neurons of the La Manno dataset. These observations across datasets indicate that the CellCover marker gene panels from the Telley data accurately define the primary cell states traversed during neocortical neurogenesis in the mouse, as well as the significant heterogeneity in the transition between the progenitor and neuron states.

To explore if these gene panels define neocortical cell types conserved across species in mammalian neurogenesis, we also examined expression of the panels derived from the mouse in scRNA-seq data from human mid-gestational cortical tissue (Polioudakis et al., 2019; Figure 2D and E). Markers of 1 H mouse cells are most frequently and specifically expressed in the progenitor cells of the human neocortex. Markers of 24 H cells are most frequently expressed in IPCs and earliest neurons that have not migrated out of the GZ, while 96 H markers show greatest frequency in later neurons resident in the CP. This clear mapping of the murine CellCover markers onto human cortical data demonstrates that these gene panels capture neurogenic cell states conserved across mammalian development.

**Figure 3.**
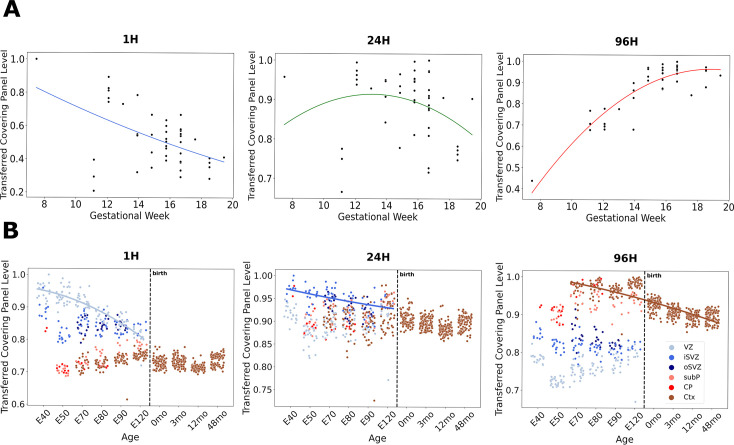
Expression of 1 H, 24 H, and 96 H CellCover marker gene panels across development in bulk human and microdissected macaque neocortical tissue. (**A**) Transfer of CellCover marker gene panels from the Telley data in mouse (Telley et al., 2019) into bulk RNA-seq from human fetal cortical tissue (Jaffe et al., 2018). The panel is obtained using the CellCover with $\boldsymbol{\alpha}=0.02$ and.$\boldsymbol{d}=5$ The transferred values of gene panels were assessed as the sum of panel gene expression levels in each individual sample divided by the maximum sum observed in the samples. (**B**) Transfer of marker gene panels (Appendix 4—table 1d: $\boldsymbol{\alpha}=0.02$, $\boldsymbol{d}=15$ nested expanded from $\boldsymbol{d}=7$) from the Telley data in mouse into microarray data from laser microdissected regions of the developing macaque neocortex (Bakken et al., 2016). Nonlinear fits in 1 H, 24 H, and 96 H panels are to VZ, iSVZ, and Ctx data, respectively. X-axis ages are expressed as embryonic (E) days after conception and months (mo) after birth. Transferred marker gene panel levels were calculated as in panel A. VZ = ventricular zone, iSVZ = inner subventricular zone, oSVZ = outer subventricular zone, subP = subplate, CP = cortical plate, Ctx = cortex.

These transfers of the Telley 1 H, 24 H, and 96 H CellCover marker gene panels into single-cell data of the mouse and human neocortex provide a cell-type-level perspective on the marker panels. To further understand the signals captured in these panels, we transferred them into additional data with much less cellular resolution but greater temporal and anatomical resolution. In bulk RNA-seq of human postmortem prefrontal cortical tissue (Jaffe et al., 2018), we observed a decrease in the expression of 1 H markers across human neocortical development in utero (Figure 3A). This is consistent with the progressive disappearance of neural progenitors as post-mitotic neurons are generated during peak neurogenesis in mid-gestation. The expression of 24 H markers appears to peak around GW12. Markers of 96 H cells increase in expression during fetal development, plateauing around GW16, consistent with the progressive accumulation of newly born neurons in the developing cortex during this period.

**Figure 4.**
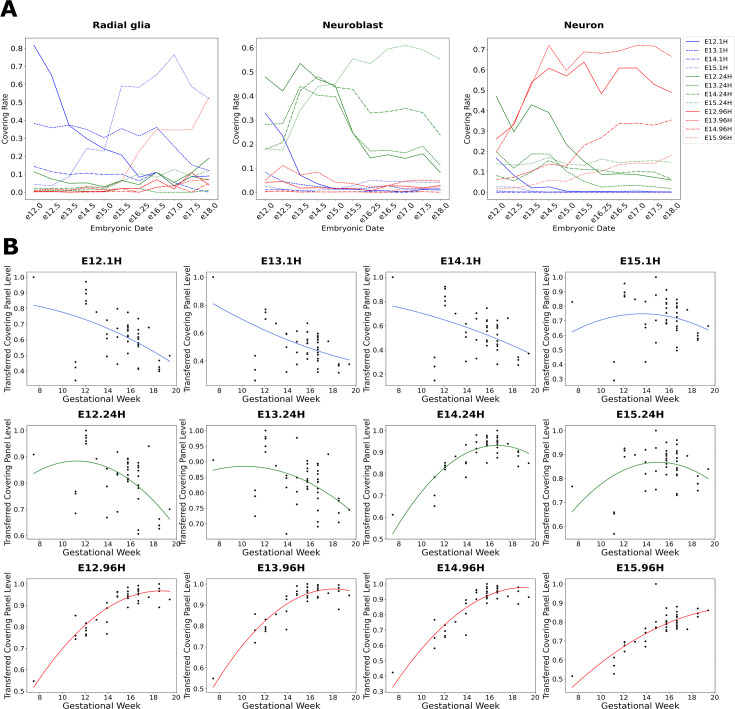
Expression of the 12 CellCover gene panels from the Telley data across development in the fetal neocortex of the mouse and human. (**A**) Transfer of Telley gene panels ($\boldsymbol{\alpha}=0.02$ and $\boldsymbol{d}=6$) into the radial glia (left panel), neuroblasts (center panel), and neurons (right panel) from the developing mouse brain atlas (La Manno et al., 2021). In all cases, covering rates of transferred panels from 1 H cells are depicted in blue, 24 H in green, and 96 H in red. Transferred levels were calculated as the proportion of cells of each type expressing more than three marker genes in the gene panel. (**B**) Transfer of the 12 gene panels ($\boldsymbol{\alpha}=0.02$ and $\boldsymbol{d}=15$ nested expanded from $\boldsymbol{d}=7$) into bulk RNA-seq data from the human fetal cortex (Jaffe et al., 2018). Transferred values of gene panels were assessed as the sum of gene panel expression levels in each individual sample divided by the maximum sum observed in the samples (as in Figure 3A).

**Figure 5.**
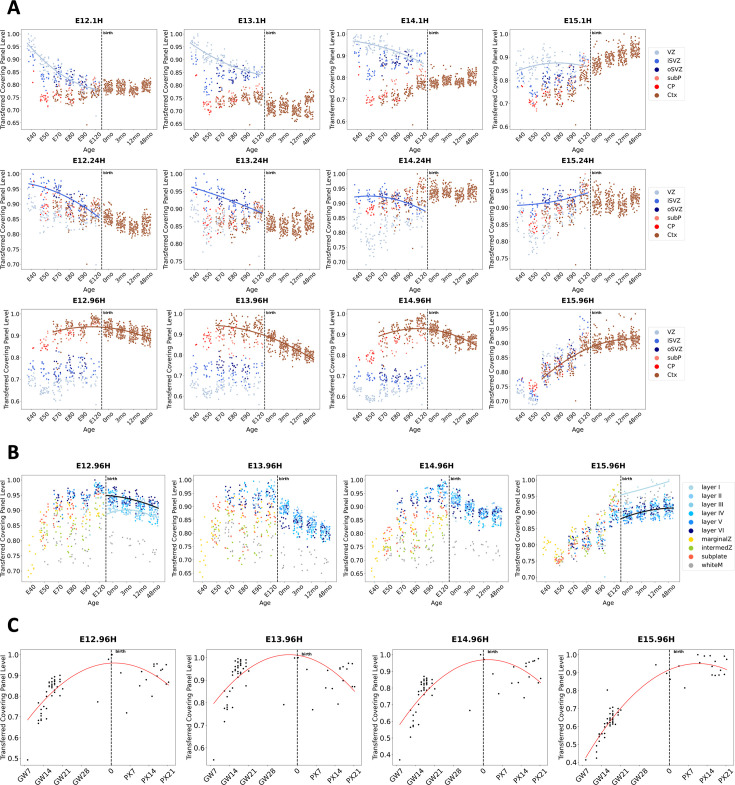
Expression of the 12 CellCover gene panels from the Telley data across development in the fetal and early postnatal neocortex of the macaque and human. The CellCover marker panels are obtained at $\boldsymbol{\alpha}=0.02$ and $\boldsymbol{d}=15$ nested expanded from $\boldsymbol{d}=7$. (**A**) Transfer of the 12 CellCover panels into microarray data from microdissected regions of the developing macaque neocortex (Bakken et al., 2016). X-axis ages are expressed as embryonic (E) days after conception and months (mo) after birth. Transferred gene panel levels were calculated as in Figure 3. VZ = ventricular zone, iSVZ = inner subventricular zone, oSVZ = outer subventricular zone, subP = subplate, CP = cortical plate, Ctx = cortex. (**B**) Repeated transfer of the 96 H gene panels into the microdissected macaque data, using additional labeling of dissections by cortical layer. (**C**) Repeated transfer of the 96 H gene panels into the human cortex data (Jaffe et al., 2018), showing additional late fetal and early postnatal samples in postnatal development.

Cell states across mammalian neurogenesis are tightly linked to the position in the developing cortex. To explore the precise positional association of signals captured by CellCover panels from the developing mouse cortex, we transferred the markers derived from the Telley data into expression data from hundreds of laser microdissected regions of the developing primate cortex (Bakken et al., 2016; Figure 3B). Markers of the 1 H mouse neural progenitor cells show the highest expression in the ventricular zone (VZ) of the macaque and decrease over fetal development (Figure 3B left panel), consistent with the widely held notion that ventricular RG are the earliest and most conserved neural progenitor state in the developing mammalian cortex. In contrast, markers of 24 H cells in the mouse are most highly expressed in the cells of the inner subventricular zone (iSVZ) of the primate (Figure 3B center panel), indicating a progression from the ventricular zone state to delaminated IPCs/basal progenitor (BPs) states, but not reaching the primate-specific outer subventricular zone (oSVZ; Betizeau et al., 2013)/oRG states (Hansen et al., 2010). Genes marking 96 H cells are most highly expressed in the postmitotic neurons of the fetal cortex and decrease following birth. Given the greater signal of the 96 H gene panel in more mature neurons in Figure 2E, we conclude that these genes are not related to transient newborn neuron states. Rather, it is likely that the increasing glial content of the postnatal neocortex begins to reduce the relative level of the neuronal signal in this bulk RNA-seq data, despite its high spatial resolution. It is of interest that in the VZ, the 1 H signal is high and falling while the 96 H signal is low but rising. These opposing dynamic signatures within mammalian neural progenitors appear to indicate that progenitor and neuron states begin to converge over development. Elements of this have been noted elsewhere (Telley et al., 2019; Chen et al., 2022) and become clearer later in this report as we perform additional transfers of marker gene panels at higher resolution (Figures 4 and 5).

**Figure 5—figure supplement 1.**
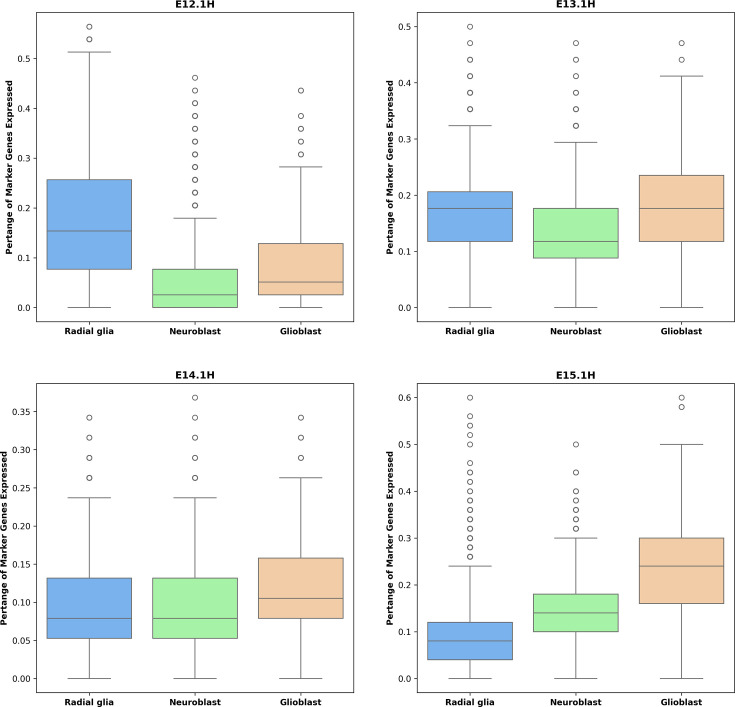
Transfer of Telley within 1 H markers to radial glia, neuroblast, and glioblast cell populations in La Manno Dataset. We use the box plot to show the distribution of the proportion of marker genes expressed in each of the three cell populations. For example, the first (blue) box plot in the top left panel shows the distribution of the proportion of E12.1H marker genes expressed in the radial glia cells in the La Manno dataset. These data clearly show the shift of mouse neocortical progenitors from neurogenic to gliogenic potential from E12-E15.

While these concise, low covering depth gene panels can effectively map conserved cell-type-specific expression dynamics across mammalian development, the multiplicity of near-optimal solutions to the covering problem (Appendix 3) suggests that a broader complementary approach for connecting more genes to cell classes will be useful to define underlying cell-type-specific mechanisms. Therefore, we proposed the CellCover nested expansion (see Nested marker set expansion in Methods) and demonstrate its utility by expanding the 1 H, 24 H, and 96 H gene panels to include additional marker genes. Specifically, nested expansion involves iteratively rerunning CellCover for a given cell class, using the previously optimal gene panel as a starting point and updating the algorithm with increased covering depth. We demonstrate that these expanded gene panels can be effectively used in gene over-representation analyses to explore cell function more deeply and capture fundamentally distinct signals from scRNA-seq data than DE methods (Appendix 4).

### CellCover marker gene panels in mouse neocortical development #2: Cell-type-specific temporal progression

Aiming to define cell-type-specific dynamics at higher resolution, we extended our previous CellCover analysis of the Telley data from the level of three cell ages (1, 24, and 96 hr) aggregated across embryonic days (E12-15) to 12 cell class labels, each representing a single cell age from one embryonic day (3 cell ages ×4 embryonic days = 12 cell classes). Figure 2—figure supplements 1–4 depict the individual genes in each of these 12 marker gene panels and the proportion of cells of each type which express them. Within each cell age (1 H, 24 H, or 96 H), there is consistent expression of marker genes across embryonic days. That is, markers for cells of one age at one embryonic day are often expressed in cells of the same age at other embryonic days. This indicates common molecular dynamics in progenitors following their terminal division at the ventricle, regardless of the embryonic day on which that division took place. These shared elements are most clear in E13 cells of all ages.

In contrast, at E12, 1 H cells are most distinct from other 1 H cells. This particularly early progenitor state is also observable in the La Manno mouse data (La Manno et al., 2021; Figure 4A) and the Bakken macaque data (Bakken et al., 2016; Figure 5A). This E12 1 H panel includes Crabp2, which marks a specific early neural progenitor state related to telencephalic regionalization in multiple primate datasets (Bakken et al., 2016; Micali et al., 2023). (This can be explored at: NeMO: CRABP2). At E14, 24 H cells begin to become distinct from 24 H cells of other embryonic ages, while 1 H and 96 H cells at E14 share dynamics across embryonic days. At E15, 24 H and 96 H cells are most distinct, paralleling the shift from deep to upper layer neuron generation (in the next section we gain more granular insight into these observations as we transfer the marker gene panels into additional primate and human data). These 12 CellCover gene panels can be explored in all the public datasets we use in this report at (NeMO: Telley 12 CellCover Panels; these can be compared to marker panels derived from DE methods: NeMO: Telley 12 Sets DE Genes).

In additional analyses, we compared the 12 CellCover marker gene panels to marker genes defined by DE methods. Consistent with our findings in blood-derived data (Figure 1B), genes identified by the two methods are highly divergent. Individual genes selected by CellCover have lower sensitivity but higher specificity than DE-defined marker genes. Because CellCover can borrow power across genes by optimizing the panel rather than individual markers, CellCover panels achieve high sensitivity despite the lower sensitivity of individual genes that it selects, yielding complementary combinations of genes to define cell classes (Appendix 5).

### Transfer of CellCover marker gene panels #2: Conserved cell-type-specific temporal progression in neocortical development

Using CellCover marker gene panels for the three principal cell ages in the Telley data, we have shown that CellCover can precisely define the major cell classes in mammalian neurogenesis across multiple scRNA-seq data sets (Figures 2 and 3). To evaluate the ability of CellCover marker gene panels to delineate temporal change within these cell types across cortical development, we have conducted similar transfer experiments using CellCover marker panels for all 12 cell classes in the Telley data as described above (Figure 2—figure supplements 1–4). Figure 4A shows the transfer of these 12 CellCover panels into the La Manno et al., 2021 mouse brain scRNA-seq atlas data (La Manno et al., 2021) using covering rate in each cell type as a measure of the strength of mapping of the gene panels (see Cell Type Mapping Across Datasets in Methods). As expected for each of the three main cell types across neurogenesis: (1) RG in the La Manno data show the highest covering rates of gene panels derived from 1 H cells in the Telley data (Figure 4A, left panel, in blue), (2) neuroblasts in La Manno have highest covering rates of 24 H markers (Figure 4A, center panel, in green), (3) neurons have the highest rates of 96 H markers (Figure 4A, right panel, in red).

Importantly, in the RG of the La Manno data, the four 1 H cell class panels spanning E12-E15 display sequentially ordered temporal patterns across developmental ages (sequential peaks of blue lines in Figure 4A, left panel). Notably, RG beyond E15 in the La Manno data begin to express neuronal (96 H) markers. This is consistent with conclusions of Telley et al., 2019 and a previous report of ours (Chen et al., 2022), which note that late neural progenitors begin to express markers of postmitotic neurons. Here, we extend this observation by showing that this is not the emergence of a non-specific neuronal identity but rather that late neural progenitors begin to express markers specifically of the later-born neurons they are about to produce (Figure 4A, left panel, E15.96H markers in the red dotted line).

While 24 H markers show highest covering rates in neuroblasts of the La Manno data (Figure 4A, center panel, in green), neurons also show notable levels of these markers (Figure 4A, right panel, in green). This is consistent with the annotation by Telley et al., 2019 of both progenitor and neuronal sub-populations within their 24 H cells. That is, 24 hr following terminal division, some neural progenitors have progressed to an early neuronal state, while others have not fully exited the progenitor state (denoted as BPs by Telley et al., 2019). It is also important to note in this analysis that Eday labels from the Telley data indicate the age of animals when cells were labeled. Hence E12.96H cells were labeled on E12 but harvested and sequenced on E16. This explains the later, and still clear, temporal progression of the Telley 96 H marker gene panels in the neurons of the La Manno data (Figure 4A, right panel, in red).

We also transferred the CellCover marker panels from the twelve Telley cell classes into bulk RNA-seq data from the developing human neocortex (Jaffe et al., 2018; Figure 4B). Levels of the 1 H gene panels from E12-14 are highest at the earliest time point (at GW8) and descend through the second trimester, indicating peak expression of these panels lies prior to the window of human cortical development studied here (Figure 4B, top row of panels). The gene panel from 1 H cells derived from E15, in contrast, peaks at GW14, indicating that the progenitor state captured in the mouse E15.1H cells is conserved as a more advanced state in the human, potentially indicating a shift to gliogenesis. Transferred expression levels of the 24 H gene panels all peak in the window of development captured in the human data, with panels from E12 and E13 peaking at GW12 before the E14 and E15 panels peak at GW16 (Figure 4B, middle row of panels). These sequential dynamics demonstrate a parallel between the temporal progression of delaminating ventricular progenitors or BPs in the mouse and human which map to specific ages in both species: the shift from E12/13 to E14/E15 BPs in the mouse corresponds to a change in human BPs that occurs between GW12 and GW16. In a trend inverse of that observed in the transfer of the 1 H gene panels, the 96 H panels show coordinated increases from GW12-16 and appear to begin to plateau after GW16 (Figure 4B, bottom row of panels). Consistent with a later peaking state, there is less plateauing of the E15.96H gene panel signal in the human data.

Transfer of the 12 mouse CellCover panels into laser microdissected tissue from the developing macaque (Bakken et al., 2016) recapitulated broad laminar trends that we observed in the transfer of the aggregated marker gene panels (1 H panels with high expression in the cells of the VZ, 24 H panels highest in the iSVZ, and 96 H panels in the cortex; Figure 3B). This more detailed twelve-panel transfer experiment also revealed conserved temporal elements of neocortical development (Figure 5A, note the progressively later shifts in nonlinear fits for the E12–E15 marker sets). Consistent with the well-known ‘inside-out’ (deep to upper layer) progression of neocortical neurogenesis, the E12.96H panel signal is highest in deep layers, while the E15.96H panel is elevated specifically in the most superficial and latest-born neurons of the cortex (Figure 5B, nonlinear fits).

Additionally, this E15.96H panel has a particularly distinct pattern, not neuron-specific like the earlier 96 H panels, but rather a pan-cellular signature of the maturing cortex (Figure 5A, far right in bottom row of panels). Transfer of the E15.96H panel into additional late fetal and early postnatal data from the bulk human neocortical study (Jaffe et al., 2018) shows that, unlike the earlier E12–E14.96H panels, expression of this panel continues to increase dramatically in the third trimester and after birth (Figure 5C). These observations may reflect the shift from neurogenic-only states at E12–E14 to the onset of gliogenesis at E15. Further indicating that E15.96H gene panel has captured the beginning of gliogenesis, levels of E12, E13, and E14 96 H gene panels are lowest in white matter, while the E15.96H panel shows high levels in this glia-rich tissue (Figure 5B, gray points). Similar to this 96 H signal at E15, the E15.1H gene panel also shows increases in cortex postnatally that are also likely attributable to the generation of astrocytes, which maintain a portion of the neural progenitor transcriptome (Figure 5A, right-most panel in the top row and Figure 5—figure supplement 1). This indicates that both the progenitor (1 H) and differentiated (96 H) cell states contain transcriptomic elements that have begun to shift from neuro- to glio-genesis at E15.

These transfers of the CellCover gene marker panels map the temporal progression of cell identities we have learned from the mouse data onto neocortical development in the primate and human. This approach to defining conserved molecular progression in shared mammalian neural progenitor populations may help elucidate how neurons sharing defining molecular, morphological, and physiological features are produced contemporaneously in the mammalian cortex. We propose CellCover coupled with transfers of this kind as a general tool in mapping cell-type-specific gene expression signals across experimental systems, developmental time, and species.

### Transfer of CellCover marker gene panels #3: evolution of outer radial glia cells

While the late neocortical progenitor switch from neuro- to glio-genesis is conserved across mammals, there have been significant cellular innovations in neocortical development since the rodent-primate divergence ∼100 million years ago (Nei and Glazko, 2002). Outer, or basal, radial glia (oRG) are a neocortical stem cell type that has been greatly amplified in the primate and human lineages and has been implicated in the neocortical and cognitive expansion observed in gyrocephalic species (Hansen et al., 2010; Fietz et al., 2010; Reillo et al., 2011). Here we examine the expression of CellCover marker gene panels derived from sorted cell types of the human fetal cortex to explore the evolution of this key cell type in cortical development, focusing on late progenitor cell types. Applying CellCover to scRNA-seq from sorted gliogenic progenitors and oRG cells of the fetal human telencephalon (Liu et al., 2023), we generated marker gene panels for these two distinct late human neocortical progenitor types. To understand their evolutionary relationship across mammals, we examined the expression of these markers across fetal development of the neocortex in human (Trevino et al., 2021), macaque (Micali et al., 2023), and mouse (Di Bella et al., 2021) scRNA-seq data (transfer of these gene panels into the many public datasets we use here can be explored online at: NeMO: Sorted Brain Cell CellCover Panels).

**Figure 6.**
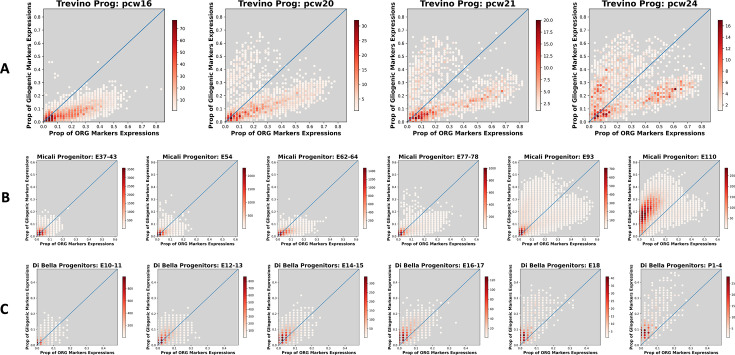
Expression of gliogenic and oRG marker gene panels across developmental time in human, macaque, and mouse neural progenitor cells. CellCover ($\boldsymbol{\alpha}=0.02,\boldsymbol{d}=15$) was used to define marker gene panels from scRNA-seq of sorted cell types of the developing human telencephalon (Liu et al., 2023). Here, the expression of marker gene panels from gliogenic precursor cells and outer radial glial (oRG) cells is examined in additional scRNA-seq data from progenitor cells of the developing (**A**) human (Trevino et al., 2021), (**B**) macaque (Micali et al., 2023), and (**C**) mouse (Di Bella et al., 2021) neocortex. Each panel depicts the number of progenitor cells (color intensity) expressing differing proportions of oRG (X-axis) and gliogenic precursor (Y-axis) marker panel genes at one developmental time in each species. Changes in the distribution of progenitor cells expressing different proportions of the marker genes as development progresses can be seen across panels from left to right. Ages are shown in individual panel titles. pcw = post-conceptional weeks.

As expected of markers of these late-stage progenitors, the proportion of markers expressed in progenitors rose across developmental time in macaque and human progenitors (Figure 6A and B). In both primate and human progenitors there are two clear cell populations which express these genes: one with expression of more oRG markers, and a later population with expression of more gliogenic markers (See Figure 6—figure supplements 1 and 2 for expression of these two marker panels in human and macaque progenitor cell subtypes). This is consistent with the current understanding of the sequential emergence of these distinct cell types in the primate and human brain. The increase in expression of human oRG markers over time was also observed in progenitor cells of the mouse neocortex (Figure 6C, Figure 6—figure supplement 3), albeit at lower proportions than in primate and human progenitors. This is consistent with the previous observation of oRG-like cells at low numbers in the developing murine neocortex (Wang et al., 2011). Importantly, unlike in primates and humans where oRG markers are expressed in neural progenitors before the arrival of gliogenic progenitors (Figure 6A and B), all mouse progenitors which show expression of human oRG markers also show expression of gliogenic markers (Figure 6C, Figure 6—figure supplement 3). This is indicated by the absence of a cell population below the identity line, while there is a clear gliogenic population above the identity at later ages. This suggests that there are likely very few, if any, bona fide oRG cells in the developing mouse neocortex, and progenitors expressing human oRG markers appear late in the mouse neocortical development and are likely gliogenic. This suggests an evolutionary model in which a portion of the oRG transcriptomic program came into being within gliogenic progenitors before the rodent-primate divergence, while additional transcriptomic programs and the bona fide oRG cell type emerged thereafter. This evolutionary model is consistent with the developmental conclusion made by the authors of the human neural progenitor sorting report who assert that gliogenic precursors are likely derived directly from oRG cells (Liu et al., 2023) and also consistent with our recent identification of a partially conserved oRG transcriptomic program with gliogenic elements that we defined using joint matrix decomposition across scRNA-seq data from these same three species (Sonthalia et al., 2026).

**Figure 6—figure supplement 1.**
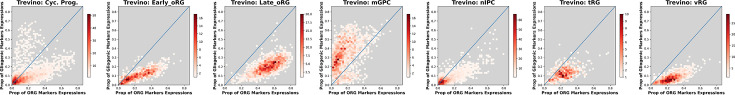
Transfer of oRG and gliogenic marker panel, derived from the sorted cell types of human fetal cortex, to human progenitor cell subtypes. The intensity of the dot at coordinate (x,y) indicates the number of human progenitor cells with $(100\times x)$ percent of oRG markers expressed and $(100\times y)$ percent of gliogenic markers expressed.

**Figure 6—figure supplement 2.**
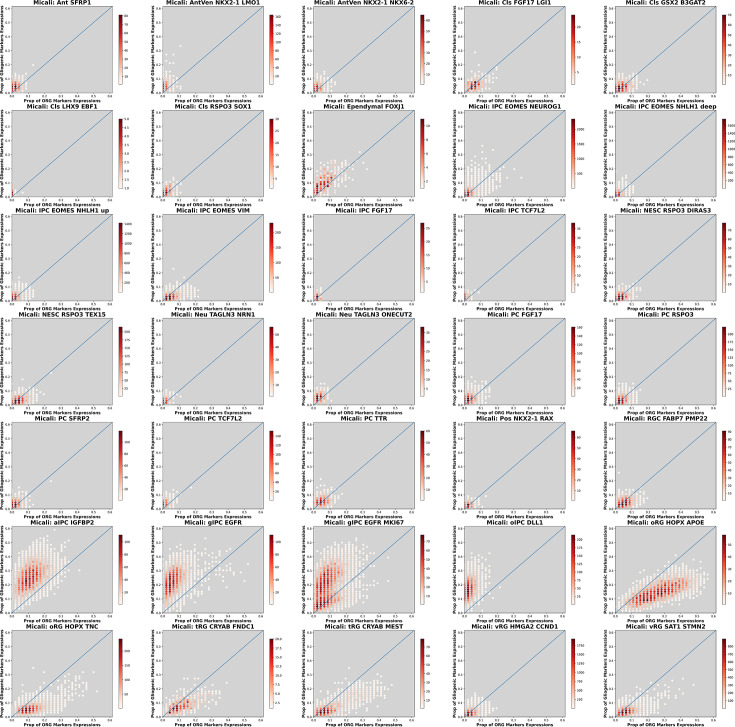
Transfer of oRG and gliogenic marker panel, derived from the sorted cell types of human fetal cortex, to macaque progenitor cell subtypes. The intensity of the dot at coordinate (x,y) indicates the number of macaque progenitor cells with $(100\times x)$ percent of oRG markers expressed and $(100\times y)$ percent of gliogenic markers expressed.

**Figure 6—figure supplement 3.**
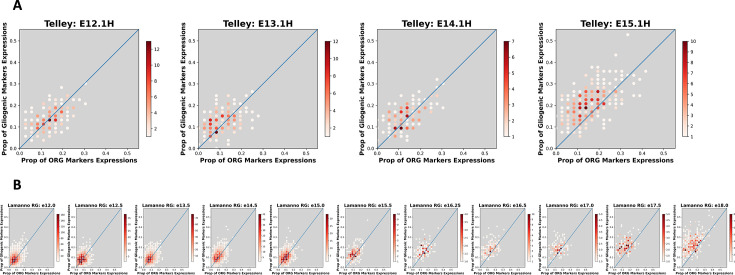
Transfer of oRG and gliogenic marker panel, derived from the sorted cell types of human fetal cortex, to mouse progenitor cells. The intensity of the dot at coordinate (x,y) indicates the number of mouse progenitor cells with $(100\times x)$ percent of oRG markers expressed and $(100\times y)$ percent of gliogenic markers expressed. (**A**) Transfer to the four embryonic dates of 1 H cells in Telley dataset. (**B**) Transfer to the La Manno radial glia cells across time.

## Discussion

In this work, we introduced CellCover, a novel algorithm designed for identifying cell-type-specific marker gene panels in scRNA-seq data. CellCover employs a minimal partial set covering approach to efficiently capture a concise set of cell type differentiating genes whose complementary expression patterns effectively distinguish cell populations. We show that marker panels selected by CellCover achieve predictive performance comparable to those identified through DE and other supervised marker selection methods. Importantly, CellCover consistently selects genes with less redundancy across cell types when compared to DE methods. In addition, although CellCover shares conceptual similarities with PhenotypeCover, as both adopt variants of the set covering problem, they differ substantially in objective, algorithmic formulations, and optimization procedures; a detailed comparison is provided in Appendix 6.

A key component of CellCover is the covering rate, which quantifies how completely a gene panel represents a cell population and, thereby, facilitates the assessment of marker gene panel generalizability across datasets. Specifically, marker panels generated from one dataset can be evaluated by their covering rate in cell populations from different datasets, enabling cross-study mapping of cell-type-specific signals. In both validation and exploration transfer experiments, we observed consistent alignment of cell types between source and target datasets along corresponding hematopoietic and neural lineages. In data from the developing mammalian neocortex, we have shown that CellCover captures cell-type-specific signals in mouse that are conserved across primate and human cortical development. Using ground truth temporal labeling of cells in the mouse cortex, we have mapped the temporal progression of murine neocortical development onto non-human primate and human developmental trajectories. Applying CellCover to sorted cell types of the fetal human cortex and transferring the resulting marker panels to mouse and primate data, we have shown that human oRG cells likely evolved through expansion of transcriptomic programs in rodent gliogenic precursors.

A limitation inherent to our method is that the partial set covering problem in the CellCover formulation is NP-hard. Consequently, integer programming solvers may experience prolonged runtimes when searching for the globally optimal solution, particularly as the required covering depth increases, for example when selecting a large gene panel. To address this issue, we introduced the nested expansion algorithm, in which the solution obtained at a smaller covering depth is carried forward as a subset of the solution at the subsequent larger depth. This iterative, nested approach significantly improves computational efficiency, especially when starting with a small initial covering depth and incrementally increasing it. We provide a runtime analysis of CellCover along with general implementation guidance in Appendix 7.

## Methods

### Computing marker sets

#### Goals

The goal of our method is to find a relatively small set of genes for each cell type that is sufficient to label each individual cell. We associate to the observation of a (random) cell of a certain tissue a random variable $X=(X_{g},g\in G)$ where $X_{g}$ denotes the raw count expression level of gene $g$. We denote the cell type labels of cells by a categorical random variable $Y\in\{1,2,\dots K\}$.

Fix a target cell type $k$. We will declare that a set of genes $M$ provides a good marker set for this cell type if (1) with high probability, a sufficient number of genes in $M$ are expressed if the cell type is $k$, and (2) genes in $M$ are expressed with low probability if the cell type is not $k$. We note that specifying $M$ is equivalent to specifying a family of binary variables $z=(z_{g},g\in G)$, taking $M=\{g:z_{g}=1\}$.

We will dedicate most of this discussion to the formulation of optimization problems that quantify conditions (1) and (2). We note that these conditions differ from what is most often accepted as a definition of a marker gene, a gene which is expressed with a significantly larger probability in type $k$ than in any other types, or, alternatively, a gene that is differentially expressed. Such criteria define markers at the individual gene level. However, our definition of *marker sets* relies on the collective behavior of the genes selected in $M$.

We now make more precise our definition of marker sets, which will take us to an integer linear problem (ILP).

#### Binarization

We define a gene *g* to be expressed if and only if $X_{g} > 0$. This operation can be refined by replacing the zero threshold by some low activity number *θ* (or $\theta_{g}$: $X_{g} > \theta_{g}$). Given this binarization rule, we introduce the random variable $U_{g}$ to encode the binary expression level of gene $g$, where

$U_{g}=\begin{cases}1\quad\text{if }X_{g} > 0\\ 0\quad\mathrm{otherwise}\end{cases}$.

#### First condition

We now formulate condition (i) as a constraint on the set $M$ and introduce two parameters of our method, namely an integer $\boldsymbol{d}$ (or ‘depth’) and a scalar $0\leq{\boldsymbol{\alpha}}\ll 1$ to quantify this condition as ‘the probability that at least $d$ genes in $M$ are expressed given that the cell type is $k$ is larger than $1-\boldsymbol{\alpha}$’, or

(1) $$\displaystyle   P\bigg(\sum_{g\in M}U_{g} \geq \boldsymbol d\ \Big|\ Y=k\bigg) \geq 1 -\boldsymbol \alpha.$$

The left-hand side of the inequality (Equation 1) is the rate at which the set $M$ covers the class $k$, or covering rate. Obviously, this constraint can be satisfied for some $M\subset G$ if and only if it is satisfied for $M=G$, so that $\boldsymbol{\alpha}$ needs to be chosen such that

$$\displaystyle  P\left(\sum_{g\in G}U_{g}\geq\boldsymbol{d}\Big{|}\ Y=k\right)\geq 1-\boldsymbol{\alpha}.$$

Note also that, using the notation introduced in the previous section,

$$\displaystyle  \sum_{g\in M}U_{g}=\sum_{g\in G}U_{g}z_{g}.$$

This condition can also be interpreted in terms of a classification error. Indeed, define $\Phi_{M,\boldsymbol{d}}$ as the binary classifier equal to one if $\sum_{g\in M}U_{g}\geq\boldsymbol{d}$ and to 0 otherwise. Then (Equation 1) expresses the fact that this classifier’s sensitivity is at least $1-\boldsymbol{\alpha}$.

#### Second condition

Having expressed the first condition as a constraint, we now express the second one as a quantity $M\mapsto F(M)$ to optimize. Ideally, one would like to minimize the probability that, conditionally to $Y_{k}=0$, there exists at least one active gene in $M$, namely

$$\displaystyle  P\bigg{(}\sum_{g\in M}U_{g}\geq 1\ \Big{|}\ Y\neq k\bigg{)}.$$

This quantity, however, is not straightforward to minimize, but its Bonferroni upper bound

$$\displaystyle  F_{0}(M)=\sum_{g\in M}P(U_{g}=1\mid Y\neq k)=\sum_{g\in G}P(U_{g}=1\mid Y\neq k)z_{g}$$

is linear in the binary vector $z$ and will be amenable to an ILP formulation. ($F_{0}(M)$ is also equal to the expectation of $\sum_{g\in M}U_{g}$ given $Y\neq k$.)

Note that, obviously

$$\displaystyle  P\bigg{(}\sum_{g\in M}U_{g}\geq r\ \Big{|}\ Y\neq k\bigg{)}\leq P\bigg{(}\sum_ {g\in M}U_{g}\geq 1\ \Big{|}\ Y\neq k\bigg{)}\leq\sum_{g\in G}P(U_{g}=1\mid Y \neq k)z_{g}$$

so that $1-F_{0}(M)$ controls the sensibility of the previous classifier $\Phi_{M,r}$.

If the variables $U_{g}$ are independent (conditionally to $Y$), then

$$\displaystyle  P\bigg{(}\sum_{g\in M}U_{g}\geq 1\ \Big{|}\ Y\neq k\bigg{)}=1-\prod_{g\in M}(1 -P(U_{g}=1\mid Y\neq k)),$$

which suggests using

$$\displaystyle  F_{1}(M)=-\sum_{g\in M}\log(1-P(U_{g}=1\mid Y\neq k))$$

as an alternative objective.

#### Margin weights

More generally, we will consider objective functions $F$ taking the form

$$\displaystyle  F(M)=\sum_{g\in G}w_{g}z_{g}$$

for some choice of weights $(w_{g},g\in G)$. The function $F_{0}$ above uses $w_{g}=P(U_{g}=1\mid Y\neq k)$, and $F_{1}$ uses $w_{g}=-\log(1-P(U_{g}=1\mid Y\neq k))$, but other choices are possible. We will in particular work with weights reflecting the ability of the variable $U_{g}$ to identify class $k$, such as

(2) $$\displaystyle   w_{g}= \frac{\sum_{l\neq k}P(U_{g}=1\mid Y = l)/(K-1)}{P(U_{g}=1\mid Y = k)}. $$

or

(3) $$\displaystyle   w_{g}= \frac{\max_{l\neq k}P(U_{g}=1\mid Y = l)}{P(U_{g}=1\mid Y = k)}.$$

where $K$ is the total number of classes. Small values of $w_{g}$ reflect a large sensibility of the variable $U_{g}$, when differentiating type $k$ from any other type. In our algorithm, we implement Equation 2 as the default while offering users the flexibility to choose their desired weight schemes. Going further, it is natural to eliminate genes for which one of the ratios above is larger than 1, which is achieved by enforcing $z_{g}=0$ if $w_{g} > 1$ (or, equivalently, replacing $w_{g}$ by $+\infty$ when $w_{g} > 1$). This choice has, in addition, the merit of reducing the number of free variables $z_{g}$ and accelerating the optimization process. Note that these margin weights ensure that $F(M)\geq F_{0}(M)$ and therefore also control sensibility.

#### Marker set estimation algorithm

We can now conclude our discussion with a description of our algorithm. Assume we observe the raw count single-cell gene expression data of $N$ cells $(x_{g}^{(c)},g\in G)$ and their cell types $y^{(c)}$ for $c=1,\ldots,N$. We binarize $(x_{g}^{(c)},g\in G)$ to obtain the binary expression levels $(u_{g}^{(c)},g\in G)$ for $c=1,\ldots,N$ as the input for our algorithm. Let $S_{k}$ be the set of cells with type $k$ for $k=1,2,...,K$. Given the hyperparameters **d** and **α**, the following algorithm returns a covering marker panel for cell type $k$, with covering depth $\boldsymbol{d}$ and covering rate $1-\boldsymbol{\alpha}$:

1. Estimate the probability that gene $g$ is expressed under cell type $l$ with

(4) $$\displaystyle   m_{g,k}= \frac{\sum_{c \in S_k}u_{g}^{(c)}}{|S_{k}|}$$

for $g\in G$ and $k=1,\ldots,K$ based on training data.

2. Compute weights $w_{g},k,g\in G$ as the ratio between the average probability of expression of $g$ when cell type is not $k$ and the probability of gene $g$ expressed under cell type $k$ (or use user-supplied weights).

(5) $$\displaystyle  w_{g,k}=\frac{\sum_{l\neq k}m_{g,l}/(K-1)}{m_{g,k}}$$

A small value of $w_{g,k}$ indicates that gene $g$ is discriminative of cell type $k$.

3. To form the candidate gene set $G_{k}$, filter out the genes that are either not discriminative or too lowly expressed, letting $G_{k}=\{g:w_{g,k}\leq 1\}\cap\{g:m_{g,k}\geq 0.1\}$.

4. Use an integer programming software (Gurobi Optimization, 2022) to minimize, with respect to binary variables $z_{g},g\in G_{k}$ and $s^{(c)},c\in S_{k}$, the function $\sum_{g\in G_{k}}w_{g,k}z_{g}$ subject to the constraints

$$\displaystyle  \sum_{g\in G_{k}}z_{g}u_{g}^{(c)}\geq\boldsymbol{d}s^{(c)},\quad c\in S_{k}$$

and

$$\displaystyle  \sum_{c\in S_{k}}s^{(c)}\geq(1-\boldsymbol{\alpha})|S_{k}|.$$

where

- $z_{g}$ is the marker gene indicator variable such that $z_{g}=1$ if gene $g$ is selected to the marker panel $M_{k}$.
- $s^{(c)}$ is the cell covered indicator variable such that $s^{(c)}=1$ if there are greater than or equal to **d** genes in $M$ being expressed in cell $c$.

5. Return the covering panel $M_{k}=\{g\in G_{k}:z_{g}=1\}$.

### Benchmark analysis

#### Gene weight computation based on binary and log-normalized expression profile

In the benchmark analysis, CellCover employs two weighting schemes for gene selection. The first uses the default weight defined on the binarized gene expression data (Equation 4 in Methods Section Marker set estimation algorithm), while the second modifies this approach by using log-normalized gene counts in place of binary expressions:

$$\displaystyle  w_{g,k}^{*}=\frac{\sum_{l\neq k}m^{*}_{g,l}/(K-1)}{m^{*}_{g,k}}$$

(6) $$\displaystyle   m^{*}_{g,k}= \frac{\sum_{c \in S_k}x_{g}^{(c)}}{|S_{k}|} $$

where $x_{g}^{(c)}$ is the log-normalized expression of gene $g$ in cell $c$. In both cases, the covering constraints are consistently defined using the binarized data. These two weighting schemes were implemented to ensure fair comparisons with other methods, as RankCorr, scGeneFit, PhenotypeCover, and DE all use log-normalized count data as input by default. Throughout the paper, unless otherwise specified, we use binary data for both weight calculation and covering constraints in CellCover.

#### Support vector machine

To evaluate the performance of marker genes identified by different methods, we employed a SVM classifier using the *sklearn.svm.SVC*() function from the scikit-learn library (Pedregosa et al., 2011). SVMs are supervised learning models that construct optimal hyperplanes in high-dimensional spaces to effectively separate multiple classes with maximum margin. To address class imbalances in our multi-class gene expression data, we applied class weight normalization by setting the *class_weight* parameter to }}*balanced′′*. This approach assigns weights to each class inversely proportional to their frequency, ensuring equal representations of different cell types during the training process. The SVM was trained exclusively on gene expression data restricted to the selected marker genes, allowing us to assess the discriminative power of these markers in accurately predicting class labels across different methods.

#### Balanced accuracy

Balanced accuracy was employed as the primary performance metric to mitigate the effects of class imbalance in our multi-class classification. Unlike conventional accuracy, which may overemphasize performance in the more prevalent classes, balanced accuracy measures the average recall (also referred to as sensitivity) across all classes. Specifically, for each class, the true positive rate is calculated, and these rates are then averaged over all classes.

#### Intersection of global marker panels

We compare each method’s global marker panel to the global marker panel produced by CellCover (log-normalized). Specifically, for a given method, we count the number of genes that overlap with the CellCover (log-normalized) global marker panel and then divide by the total number of genes in the CellCover (log-normalized) global panel. This ratio yields the proportion of intersection between each method’s global marker panel and the CellCover (log-normalized) reference panel.

#### Within marker panel redundancy

We further assess the redundancy of marker genes within the global marker panel identified by each method $x\in\{\mathrm{CellCover-LogNorm},\mathrm{DE}\}$. For each cell type $k\in{1,\ldots,K}$, we obtain a local marker panel $M_{k}^{x}$ of the same size. We then define the global marker panel $M^{x}$ as the union of all cell-type-specific marker panels

$$\displaystyle M^{x}=\cup_{1:K}M_{k}^{x}.$$

Next, let $R^{x}\in M^{x}$ be the set of genes appearing in at least two different local marker panels. The proportion of redundant genes in $M^{x}$ is thus

$$\displaystyle \frac{|R^{x}|}{|M^{x}|}.$$

This measure reflects how frequently the same gene is identified as a marker for multiple cell types by a given method.

### Cell type mapping across datasets

To quantify the consistency of mapping between cell types in the source and target datasets using the CellCover marker gene panels, we calculated the proportion of cells in each target cell type expressing at least five markers in the CellCover marker panel for each source cell type. We then applied 0–1 normalization to the covering rates of $M_{k}$ across all available cell types $k^{\prime}$ in the target dataset. This normalization was performed to standardize the covering rates of the marker panel $M_{k}$, ensuring uniform comparability across all target cell types for each source cell type marker panel. To establish a mapping between cell types in the source and target datasets, we required the normalized covering rate to exceed a threshold of 0.8.

### Nested marker set expansion

Step (4) can be modified to estimate a covering as a superset of a previous one (obtained e.g. with a smaller depth): Assume that $(z^{(0)}_{g})\in\{0,1\}^{|G_{k}|}$ are given, for example from a previous run of our algorithm, defining an initial marker panel. In order to extend this panel to a covering at depth $\boldsymbol{d}$, one only needs to replace step (4) by the following:

(4’) Use an integer programming software to minimize, with respect to binary variables $Z_{g},g\in G_{k}$, $s^{(c)},c\in S_{k}$, the function $\sum\limits_{g \in G_{k}}{W_{g,k}Z_g} $ subject to the constraints

$$\displaystyle z_g\geq z^{(0)}_g, g\in G_k$$

$$\displaystyle  \sum\limits_{g \in G_k}{z_gu^{(c)}_g}\geq {\boldsymbol ds}^{(c)}, c\in S_k $$

and

$$\displaystyle  \sum\limits_{c\in S_k}{S^{(c)}}\geq (1-\boldsymbol \alpha ) \left|S_k\right| .$$

### Remarks

- Even though it was not designed with this intent, our marker set estimation algorithm can be interpreted as an asymmetric classification method to separate type $k$ from all other types. In an approach akin to classical statistical testing, we determine a classifier with some free parameters (here, the gene set $M$) that we require to have a minimal sensitivity, $1-\boldsymbol{\alpha}$ (so that **α** is reminiscent of a type-I error). The free parameters are then optimized in order to minimize an objective function that controls (among other things) the sensibility of the classifier.
- If we take $w_{g}=1$ for all $g$, the previous algorithm only depends on training data associated with cell type $k$. The resulting algorithm provides a *minimal* gene set that covers the considered population, in the sense that all cells in that population (except a fraction **α**) have at least $\boldsymbol{d}$ active genes in the selected set. With a suitable definition of what is meant by being active, this covering algorithm was introduced in Ke et al., 2021 and applied to the determination of important gene motifs in a population of tumor cells associated with a specific phenotype.

## Figures and Tables

**Appendix 1—figure 1.**
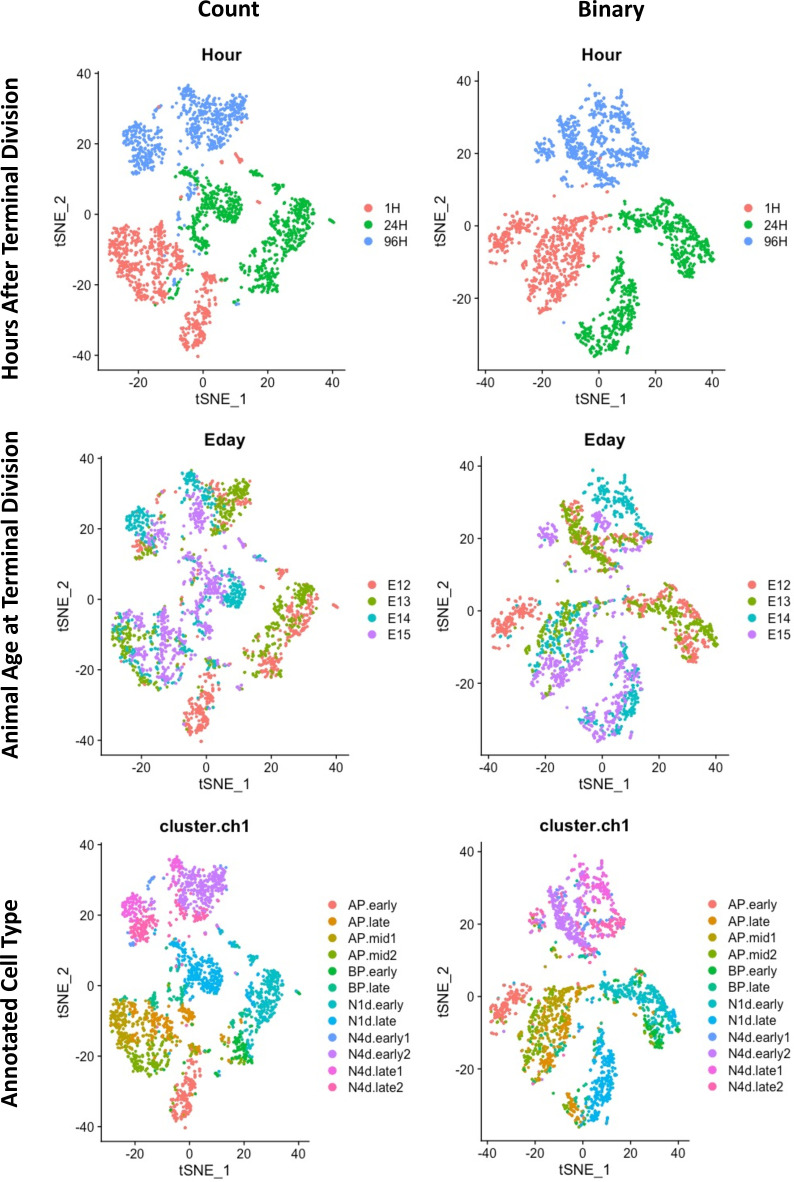
Comparison of raw count and binarized scRNA-seq data in standard unsupervised dimension reduction using the Telley (Telley et al., 2019) dataset where ventricular neural progenitor cells were labeled specifically during their final cell division and collected cells for sequencing. To focus on precise cell types, only cells of the excitatory glutamatergic neuronal lineage were included in this analysis.

**Appendix 2—figure 1.**
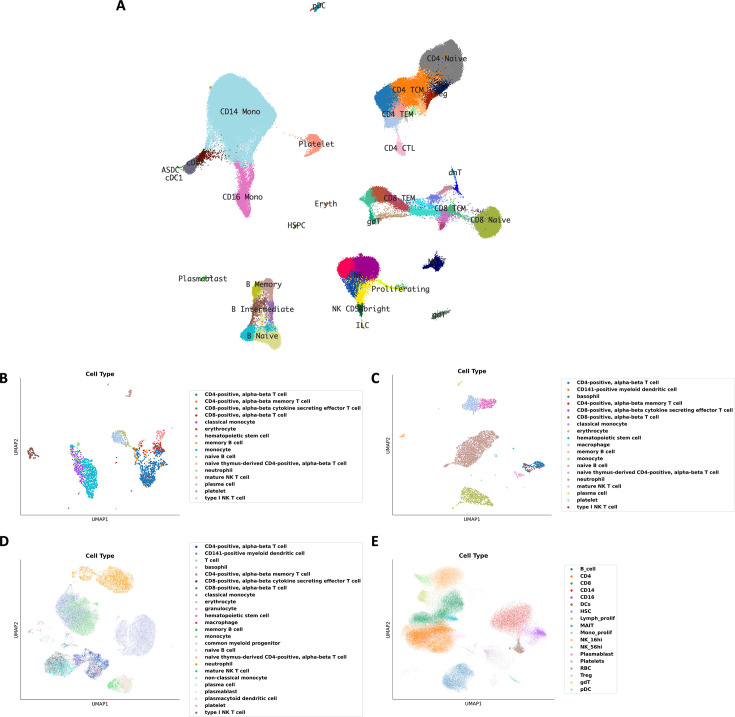
UMAP visualizations of cell type annotations for the Hao PBMC dataset, the Tabula Sapiens blood datasets, and the Stephenson COVID-19 PBMC dataset. (**A**) Hao PBMC Dataset where the UMAP visualization is available at https://atlas.fredhutch.org/nygc/multimodal-pbmc/. (**B**) Tabula Sapiens blood dataset sequenced using the Smart-seq2 protocol. (**C**) Tabula Sapiens blood dataset sequenced using the 10 x Genomics 5’ protocol. (**D**) Tabula Sapiens blood dataset sequenced using the 10 x Genomics 3’ protocol. (**E**) Stephenson COVID-19 PBMC dataset.

**Appendix 2—figure 2.**
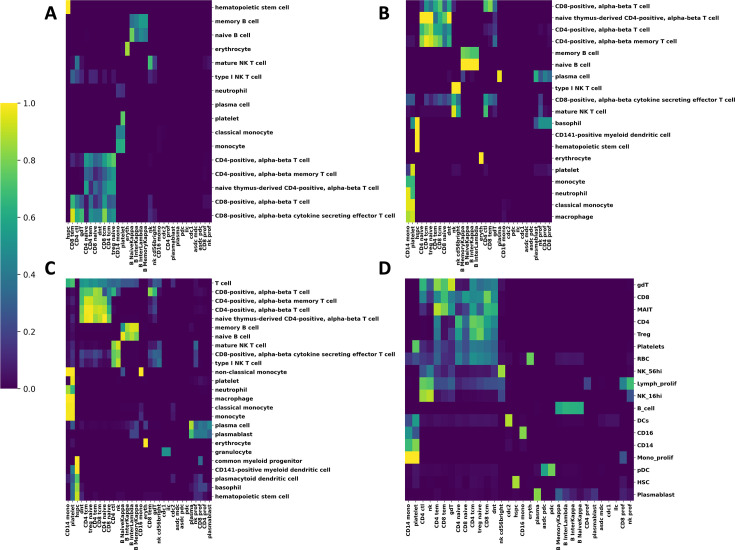
Cross-dataset marker gene transfer results. Heatmaps show the proportion of target cells (y-axis) expressing at least five markers from source cell-type-specific marker panels (x-axis) generated by CellCover. Subplots represent transfer results to (**A**) Tabula Sapiens blood datasets sequenced with Smart-seq2, (**B**) 10x5’, (**C**) 10x3’, and (**D**) the Stephenson COVID-19 PBMC dataset with only the healthy controls.

**Appendix 2—figure 3.**
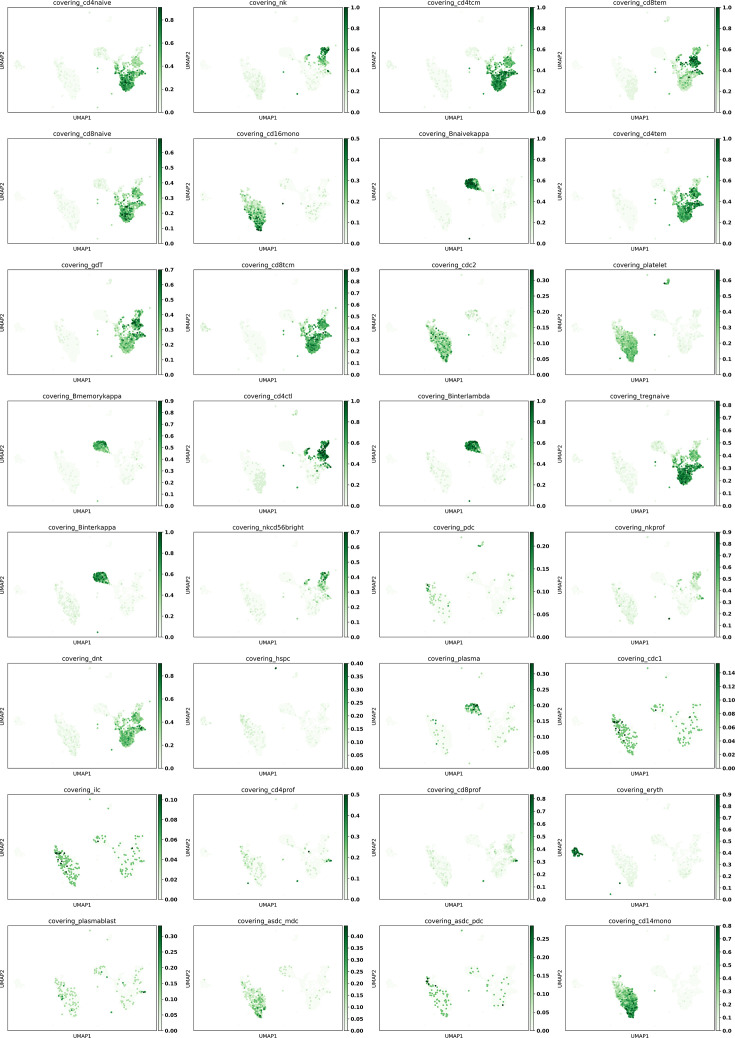
Transfer of CellCover marker panels from PBMC CITE-seq Dataset to Tabula Sapiens blood dataset sequenced by Smart-seq2 protocol. For each of the 32 cell types in the PBMC datasets, a CellCover marker panel of depth 5 and covering rate of 98% was derived. Each panel was transferred to the target dataset, with the plots showing the proportion of marker genes expressed in cells of the target dataset, as indicated by the titles of each subplot.

**Appendix 2—figure 4.**
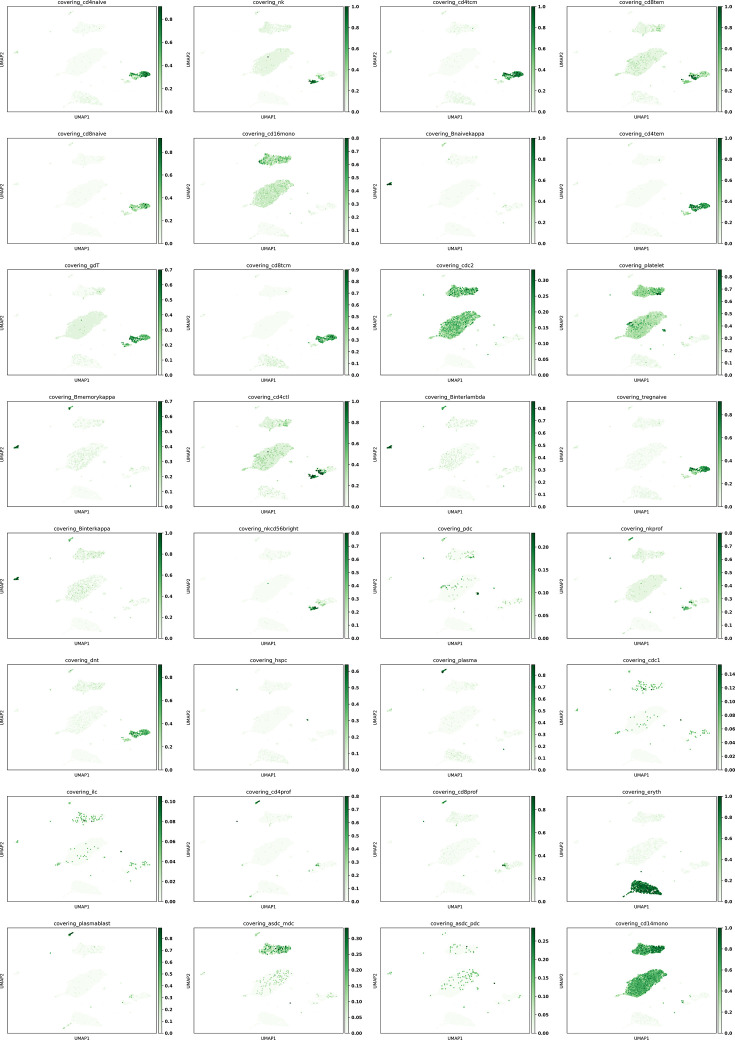
Transfer of CellCover marker panels from PBMC CITE-seq dataset to Tabula Sapiens blood dataset sequenced by 10 x Genomics 5’ protocol. For each of the 32 cell types in the PBMC datasets, a CellCover marker panel of depth 5 and covering rate of 98% was derived. Each panel was transferred to the target dataset, with the plots showing the proportion of marker genes expressed in cells of the target dataset, as indicated by the titles of each subplot.

**Appendix 2—figure 5.**
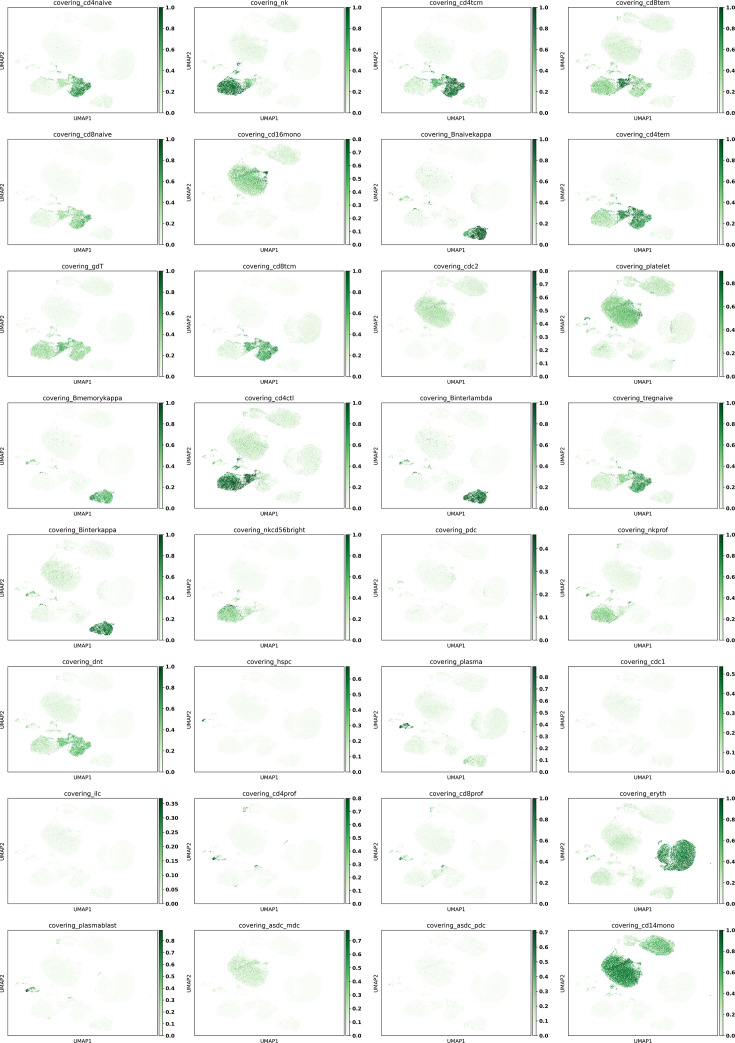
Transfer of CellCover marker panels from PBMC CITE-seq dataset to Tabula Sapiens blood dataset sequenced by 10 x Genomics 3’ protocol. For each of the 32 cell types in the PBMC datasets, a CellCover marker panel of depth 5 and covering rate of 98% was derived. Each panel was transferred to the target dataset, with the plots showing the proportion of marker genes expressed in cells of the target dataset, as indicated by the titles of each subplot.

**Appendix 2—figure 6.**
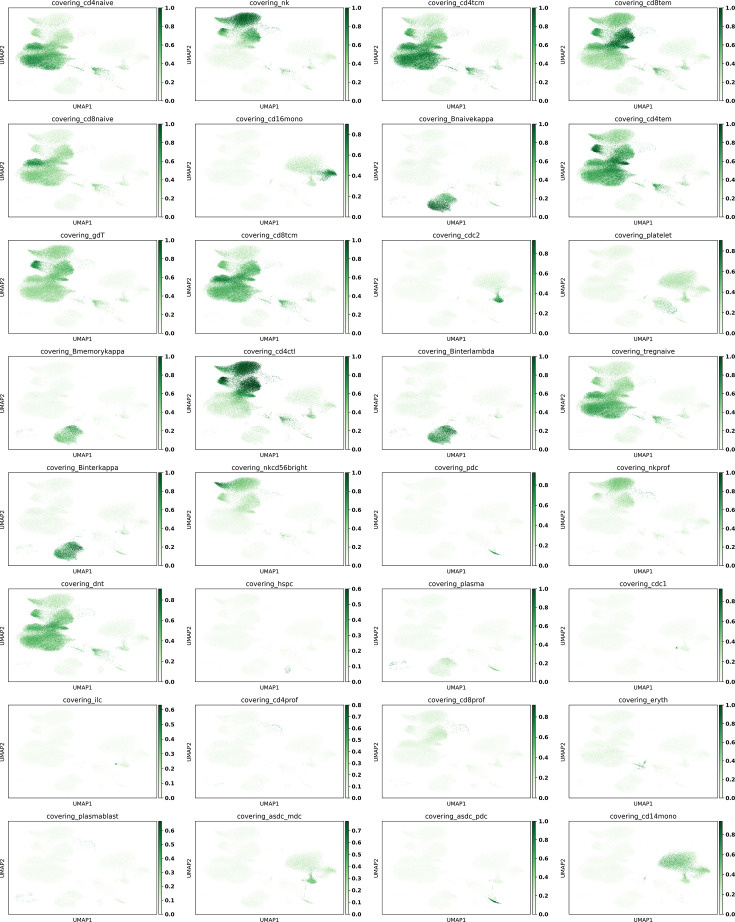
Transfer of CellCover marker panels from PBMC CITE-seq dataset to Stephenson COVID-19 PBMC dataset. For each of the 32 cell types in the PBMC datasets, a CellCover marker panel of depth 5 and covering rate of 98% was derived. Each panel was transferred to the target dataset, with the plots showing the proportion of marker genes expressed in cells of the target dataset, as indicated by the titles of each subplot.

**Appendix 3—figure 1.**
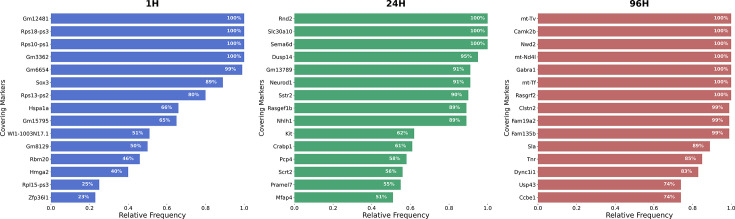
Telley CellCover marker genes with highest relative frequency. This bar plot shows the top cell age covering markers ordered by the relative frequency of each gene being selected by the covering algorithm with $\boldsymbol{d}=5$, $\boldsymbol{\alpha}=0.02$ on the Telley dataset.

**Appendix 3—figure 2.**
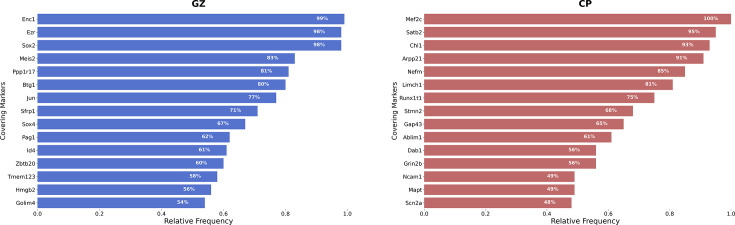
Geschwind CellCover marker genes with highest relative frequency. The bar plot shows the top cell age covering markers ordered by three relative frequency of each gene being selected by the covering algorithm with $\boldsymbol{d}=3$, $\boldsymbol{\alpha}=0.05$ on the Geschwind dataset.

**Appendix 4—figure 1.**
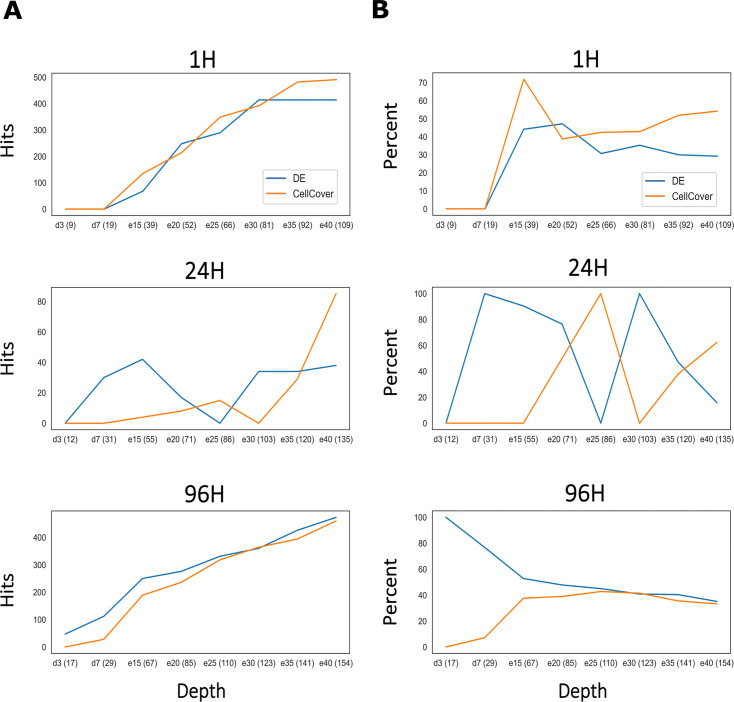
Over-representation analysis in gene marker panels of differing size and methodology, using the GO database. Over-representation analysis was performed using the Fisher’s exact test using gene sets from the GO database. Over-represented gene sets were marked as significant by a $FDR < 0.1$ cutoff using the Benjamini-Hochberg method. The background gene list was defined as all the genes present in the Telley dataset. (**A**) The number of GO gene sets found to be significantly enriched in the gene marker panels derived from CellCover and DE methods across different matched panel sizes. The X-axis indicates the depth parameter d that was set for the CellCover run (preceding the numbers on the X-axis, ‘d’ indicates the depth in de novo CellCover runs, ‘e’ indicates expanded runs that use marker panels from the preceding CellCover run as a starting point). Corresponding numbers of DE markers were selected for each depth so that marker panels of equal size were compared across CellCover and DE methods. Numbers in parentheses indicate the number of genes in each marker panel. (**B**) The proportion of gene sets that were found to be significant by one method and not the other.

**Appendix 5—figure 1.**
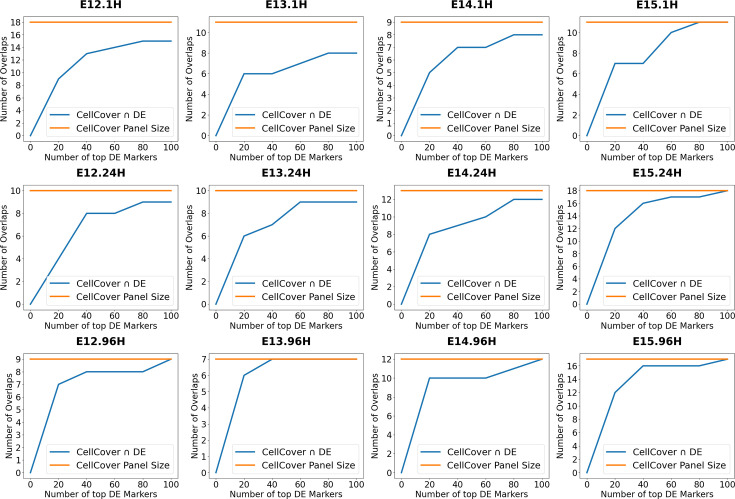
Overlap and discordance between marker genes selected by CellCover and DE methods. For each of the 12 CellCover gene panels defined in the Telley mouse data, the number of overlapping genes found by DE methods is quantified. In each panel, the orange line indicates the number of genes in the CellCover gene panel for that cell class. The blue line indicates the cumulative number of CellCover genes also found in the top $x$ ranked DE gene marker list as increasingly large DE gene lists are considered. The X-axis value where the orange and blue lines meet indicates the number of DE markers that would need to be selected to include all the CellCover markers.

**Appendix 5—figure 2.**
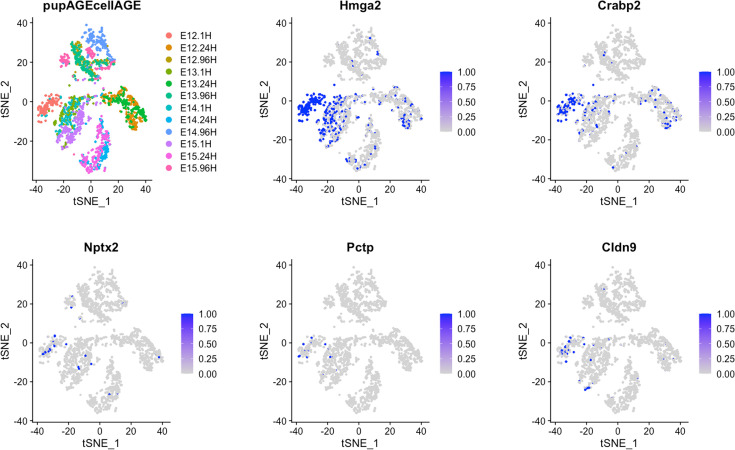
Sensitivity and specificity of individual marker genes selected by CellCover. Visualization of expression of individual marker genes selected by CellCover. Among all the covering markers for E12.1H, Hmga2 and Crabp2 have the lowest p-values in DE (and are therefore selected as DE marker genes) and show high sensitivity with lower specificity. In contrast, Nptx2, Pctp, and Cldn9 have the highest p-values in DE (and are therefore not selected as DE marker genes) and show low sensitivity individually, but very high specificity.

**Appendix 5—figure 3.**
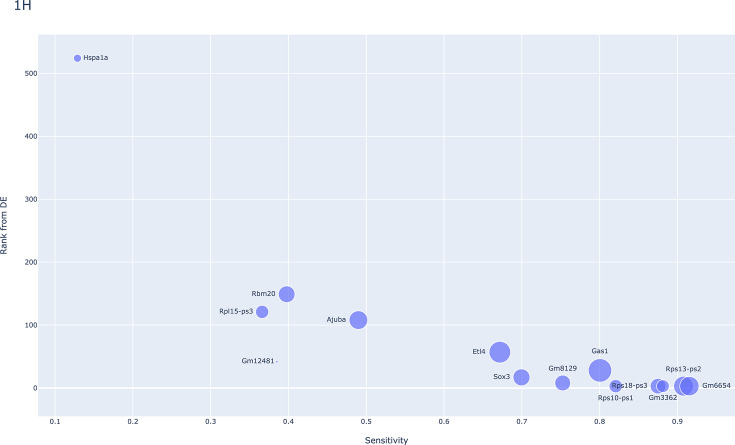
Telley 1 H CellCover markers sensitivity vs rank from DE. We ran Seurat DE on the Telley dataset to discriminate 1 H cells and rank the entire gene portfolio by the p-values. Each CellCover gene $g$ (Figure 2A) has its sensitivity of the class $k$, $P(X_{g} > 0|Y=k)$, on the horizontal axis and its rank from DE on the vertical axis. The size of the dot represents the weight of the marker defined in the method section (download the interactive plots at DE Rank vs Sensitivity). The ability of CellCover to borrow power across marker genes of low sensitivity and high specificity is one of the primary factors that sets it apart from DE methods.

**Appendix 5—figure 4.**
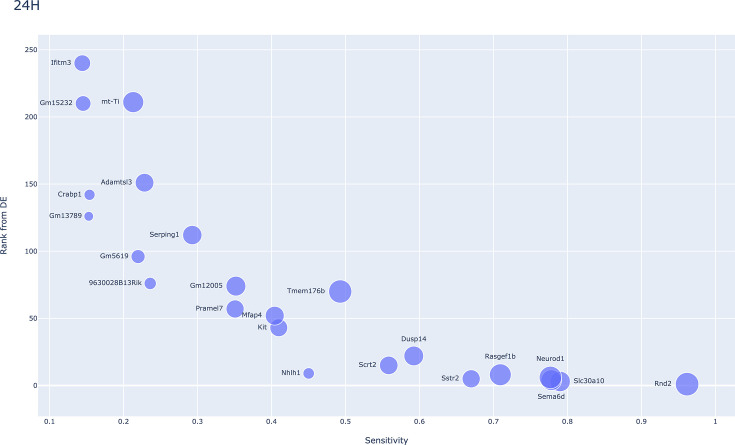
Telley 24 H CellCover markers sensitivity vs rank from DE. We ran Seurat DE on the Telley dataset to discriminate 24 H cells and rank the entire gene portfolio by the p-values. Each CellCover gene $g$ (Figure 2A) has its sensitivity of the class $k$, $P(X_{g} > 0|Y=k)$, on the horizontal axis and its rank from DE on the vertical axis. The size of the dot represents the weight of the marker defined in the method section (download the interactive plots at DE Rank vs Sensitivity). The ability of CellCover to borrow power across marker genes of low sensitivity and high specificity is one of the primary factors that sets it apart from DE methods.

**Appendix 5—figure 5.**
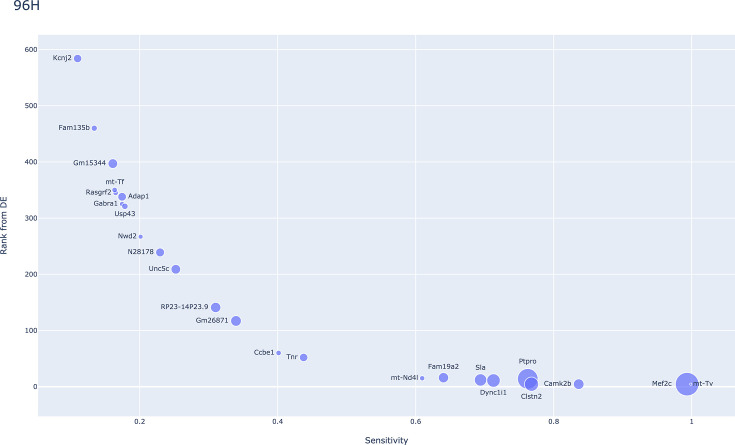
Telley 96 H CellCover markers sensitivity vs rank from DE. We ran Seurat DE on the Telley dataset to discriminate 96 H cells and rank the entire gene portfolio by the p-values. Each CellCover gene $g$ (Figure 2A) has its sensitivity of the class $k$, $P(X_{g} > 0|Y=k)$, on the horizontal axis and its rank from DE on the vertical axis. The size of the dot represents the weight of the marker defined in the method section (download the interactive plots at DE Rank vs Sensitivity). The ability of CellCover to borrow power across marker genes of low sensitivity and high specificity is one of the primary factors that sets it apart from DE methods.

**Appendix 6—figure 1.**
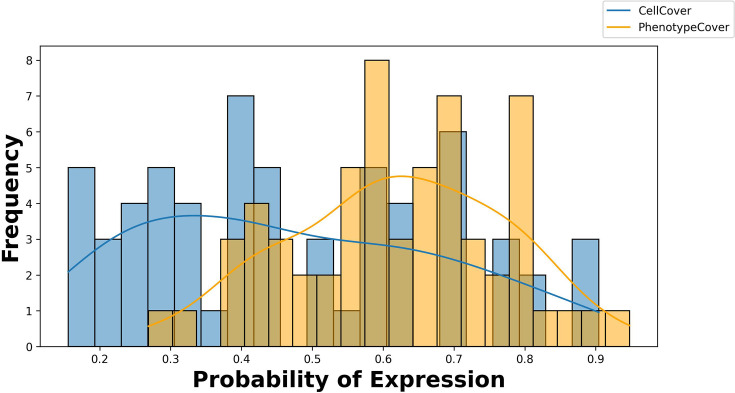
Distribution of marker gene expression rate of CellCover and PhenotypeCover. The blue bars form the histogram of the probabilities of expression of the CellCover (global version) marker genes obtained at $\boldsymbol{d}=20$ and $\boldsymbol{\alpha}=0.02$. The probability of expression is computed by $\frac{\sum_{c=1}^{N}u_{g}^{(c)}}{N}$. The yellow bars depict the distribution of probabilities of expression of the Phenotype marker gene obtained by letting $B=95$ to match the size of the CellCover marker panel.

**Appendix 7—figure 1.**
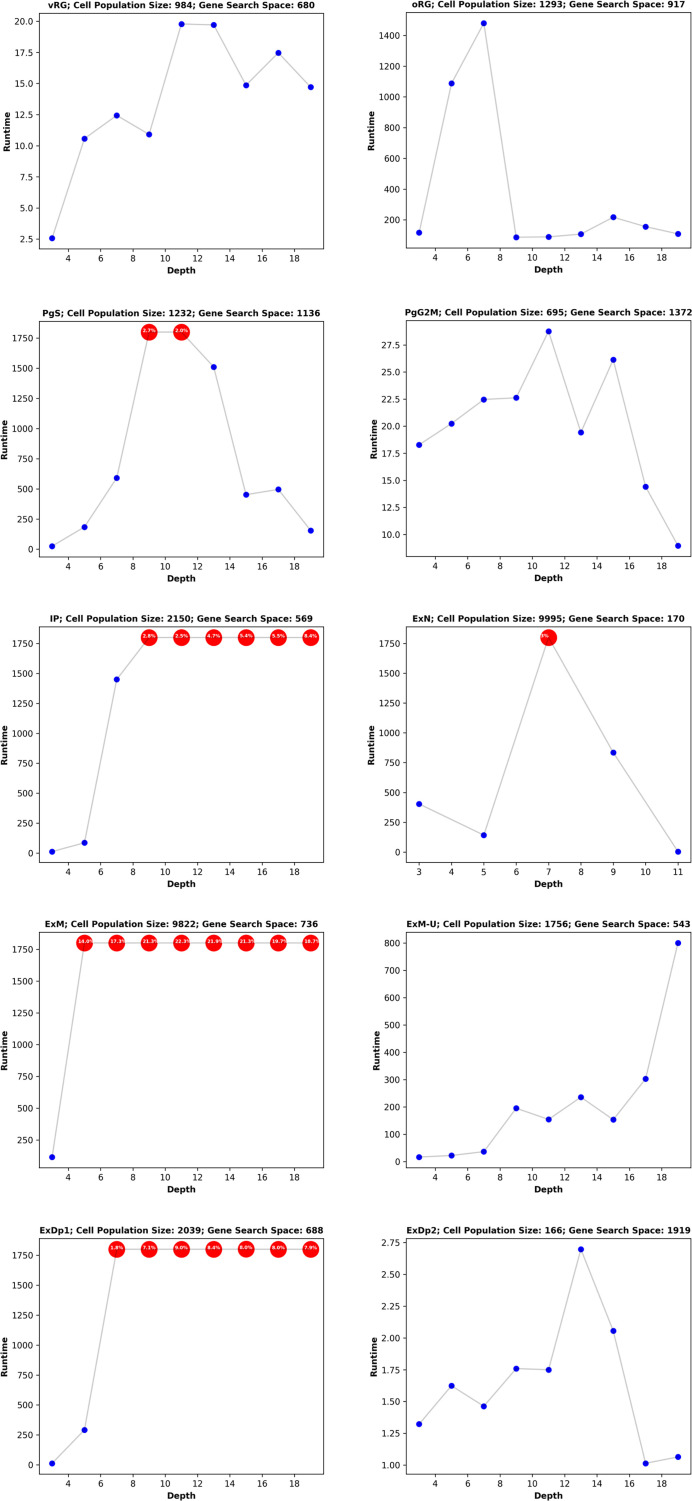
CellCover run time of multiple cell populations from the Polioudakis dataset. The $x$ axis is the covering depth $\boldsymbol{d}$, and the $y$ axis is the run time. We set a time limit of 1800 s for the program. If the branch and cut algorithm does not converge in 1800 s, MIP gap will be reported. The smaller the gap, the closer the dual and primal objectives are, indicating better convergence.

## Data Availability

While we have not generated any new data in this report, all the public RNA-seq data used in the analyses is freely explorable at NeMO Analytics (https://nemoanalytics.org/expression.html?gene_symbol=SOX2&gene_symbol_exact_match=1&is_multigene=0&layout_id=CellCover). Links to the data we used and original data sources are available at NeMO Analytics. We invite researchers to interrogate all the public data resources we examine here in the NeMO Analytics multi-omics data exploration environment that is designed to provide biologists with no programming expertise the ability to perform powerful analyses across collections of data in brain development (Sonthalia et al., 2026). Users can visualize individual genes (NeMO: Individual genes in cortex and NeMO: Individual genes in blood) or groups of genes simultaneously across multiple datasets, including automated transfer of the CellCover gene marker panels we have identified across cell types and developmental time (NeMO: Telley 3 CellCover Panels, NeMO: Telley 12 CellCover Panels, NeMO: Sorted Brain Cell CellCover Panels, and NeMO: Blood 34 CellCover Panels). CellCover is available in CellCover R and CellCover Python.

## Additional files

MDAR checklist

## Appendix 1

Binarization of gene expression data, where zero counts remain zero and positive counts map to 1, is a key step in defining minimal coverings of cell types with CellCover. This approach has been used in various contexts, including the study of cell-type-specific transcriptional dynamics (Grabski and Irizarry, 2022), imputing the count expression profile (Covert et al., 2023), and cell age prediction (Yu et al., 2023). We evaluated the effects of binarization on data exploration using the Telley dataset (Telley et al., 2019), which contains scRNA-seq data labeled by both the developmental age of the mouse pup (E12–E15) as well as the exact time since the terminal division of each individual progenitor cells (1, 24, or 96 hr). Throughout this report, we leverage this uniquely annotated dataset to explore the developmental progression of mammalian neocortical neurogenesis. These 12 cell classes (4 pup ages × 3 times since final cell division) were analyzed to determine whether binarized data could preserve class-specific expression patterns. Two methods were applied:

1. Supervised prediction of ground truth labels: Among various combinations of data types and prediction models, PCA-processed binarized data paired with a one-layer neural network achieved the highest average prediction accuracy for the 12 classes (Appendix 1—table 1). Similar results were observed in the Polioudakis dataset (Polioudakis et al., 2019), where binarized data outperformed log-transformed counts in predicting cell locations (Appendix 1—table 2).
2. Unsupervised dimension reduction and clustering: Using t-SNE or UMAP embeddings and Louvain clustering in the Telley dataset, binarized data achieved clearer separation of developmental cell classes compared to continuous counts (Appendix 1—figure 1). Quantitatively, binarized data produced higher adjusted Rand indices (ARIs), demonstrating stronger concordance between ground truth labels and clustering results (Appendix 1—table 3).

These results show that binarized gene expression data maintain sufficient structure to analyze class-specific patterns effectively.

**Appendix 1—table 1.** Average accuracies of different data types and supervised classifiers in recovering the 12 time-based cell labels in the Telley data. Subscript ‘b’ indicates binary data; all other trials used normalized log2 count level data. XGB = XGBOOST (Chen and Guestrin, 2016). ‘“PCA’ indicates using only the 1st 100 principal components. $\mathrm{NN}x$ refers to a neural network with $x$ hidden layers and 64 total nodes in the network. The size of the training set is 2342, and the size of the test set is 414.

XGB	XGB_b_	$\textbf{XGB}^{\textbf{PCA}}$	$\textbf{XGB}^{{\textbf{PCA}}}_{{\textbf{b}}}$	NN1	NN1_b_	NN2	NN2_b_	NN3	NN3_b_
95.0	93.6	86.5	91.2	93.8	**97.1**	93.3	96.4	87.9	94.9

**Appendix 1—table 2.** Comparison of binary and count data in predicative capacity in Poliousdakis dataset. Subscript ‘b’ indicates binary data; other trials used normalized log count level data. XGB = XGBOOST. ‘PCA’ indicates where classification was based on the 1st 100 principal components. = $\mathrm{NN}_{x}$ Neural net with $x$ layers, where $x$ indicates the number of hidden layers with 64 nodes in the network.

Donor	XGB	XGB_b_	$\textbf{XGB}^{\textbf{PCA}}$	$\textbf{XGB}^{{\textbf{PCA}}}_{{\textbf{b}}}$	NN1	NN1_b_	NN2	NN2_b_	NN3	NN3_b_
**368**	91.7	90.4	91.1	92.7	91.0	**93.1**	90.7	92.6	90.6	92.5
**370**	87.2	84.7	86.7	**87.8**	85.9	87.1	86.6	87.5	86.2	87.3
**371**	91.8	90.8	90.9	91.8	89.7	**91.9**	90.0	91.8	90.1	91.6
**372**	92.0	91.1	91.7	92.4	92.1	**92.8**	91.9	92.6	91.7	92.4

**Appendix 1—table 3.** Adjusted Rand Indices (ARI) for different inputs. Each entry of the table shows the ARI between Seurat clustering results and true time clusters for each version of input data (count or binary expression profile of the indicated number of genes).

	Count	Binary
**Random 2000 genes**	0.32	0.37
**Random 4000 genes**	0.34	0.39
**Random 6000 genes**	0.36	0.41
**Random 8000 genes**	0.35	0.44
**All genes**	0.37	0.45

## Appendix 2

To demonstrate that CellCover effectively captures cell-type-specific information across diverse single-cell RNA-seq datasets, we performed cross-dataset marker transfer experiments. Using Hao PBMC CITE-seq dataset (Hao et al., 2021; Appendix 2—figure 1A) as the source dataset with marker panels generated at depth 5 and a covering rate of 98%, we evaluated the transferability of CellCover markers to four target datasets (Appendix 2—figure 1B–E): Tabula Sapiens blood data (Jones et al., 2022) sequenced with three protocols (smart-seq2, 10x3’, and 10x5’) and the Stephenson COVID-19 PBMC dataset (Stephenson et al., 2021), restricted to cells from healthy controls.

For each transfer experiment, we mapped cell type $k$ in the source dataset to cell type $k^{\prime}$ in the target dataset by calculating the proportion of $k^{\prime}$ cells expressing at least five markers from the source marker panel $M_{k}$. Appendix 2—figure 2 illustrates the results of these transfer experiments. The heatmaps reveal that the marker genes identified by CellCover are primarily expressed in the corresponding cell types in the target datasets, underscoring the robustness and cell-type specificity of the method across different sequencing technologies and biological contexts.

To augment the transfer results at cellular resolution, we also show in Appendix 2—figures 3–6 the proportion of genes in the CellCover marker panel $M_{k}$ expressed in each cell from the target dataset for all cell types $k$ in the source data. These figures highlight that the cells in the target datasets with the highest abundance of $M_{k}$ expression correspond to cell states closely related to the source cell type $k$, further supporting the specificity and transferability of CellCover marker panels.

## Appendix 3

The compact marker panels generated by CellCover capture cell-type-specific signals in mouse neurogenesis within a dataset (Figure 2A), which are consistent across additional mouse data (Figure 2B) and are conserved across species when transferred to primate (Figure 3B) and human prenatal scRNA-seq data (Figures 2D and 3B). While these small sets of marker genes are useful for identifying cell classes, theoretically, there exist many unique minimal covering panels which satisfy a specific covering depth, $\boldsymbol{d}$ and false negative covering rate, **α**. To demonstrate this empirically, we used a nonparametric bootstrap approach whereby for each of the three cell ages in the Telley data, we sampled with replacement from the cells for the age of interest using a sample size equal to the number of cells in the original sample and again ran the CellCover algorithm (a conventional bootstrap design). This was repeated in each of 100 iterations with parameters identical to those used to define the original covering panels in Figure 2A. Appendix 3—figure 1 lists the 15 genes which appeared in the most covering solutions along with the fraction of solutions in which they appeared. Across the covering marker gene panels for the three cell ages, only between 3 and 7 of the top 15 genes appeared in all solutions across the 100 bootstrap iterations for the three cell classes, and several top genes appeared in <50% of solutions. Similar results were observed in a bootstrap analysis of CellCover in the Geschwind data distinguishing cells captured from the germinal zones versus cortical plate (Appendix 3—figure 2).

This multiplicity of good solutions to the covering problem in cell classification (and the cell type marker problem in general) indicates that while the small covering marker gene panels can effectively identify cell classes, a broader complementary approach for connecting more genes to cell classes will be useful in order to define underlying cell type specific mechanisms.

## Appendix 4

We propose a nested approach (see Methods) to leverage the cell type specific signals captured by CellCover in order to extend the exploration of what cells are (classes) to include what cells are doing (cell type specific functions). From a small covering panel, this will create a broader list of genes that can be used to explore cell-type specific function via gene set over-representation analysis and more complex transfer learning experiments. To expand a covering gene marker panel for a particular cell class, the CellCover algorithm is simply run again, taking the initial covering set as a starting point along with new, broader covering constraints (depth, $\boldsymbol{d}$ and proportion of uncovered cells, α) as input. Enforcing the inclusion of the previously defined covering panel in this nested run ensures the original compact list of genes for cell identity will be a subset of the expanded panel to be used in the exploration of cell function and also reduces the CellCover run time (compared to a de novo run with the same expanded constraints).

Appendix 4—table 1 lists expanded covering marker gene panels for each of the three cell ages in the Telley data that we have been exploring. The original covering gene marker panels of depth 3 were expanded to depths of 7, 15, 20, 25, 30, 35, and 40. To assess the capacity of the covering marker gene panels of differing sizes to characterize cell-type-specific function, we performed over-representation analysis using Gene Ontology (GO) on all these covering panels in addition to marker panels of equal size derived from conventional differential expression (DE) marker selection (Appendix 4—figure 1). In all cases, increasing panel sizes resulted in the detection of increasing numbers of significantly enriched gene sets. The one exception to this is in the 24 H panels where, after an initial increase, a reduction in the number of hits is observed in both methods, before hits again begin to rise with increasing panel size. Strikingly, while the number of significant hits for both methods grows with increasing panel sizes, the specific gene sets found to be enriched by the two methods are quite distinct: 40–50% of the gene sets detected by one method are not found to be significant by the other. This is consistent with our other observations indicating that the two methods of gene marker definition extract fundamentally different signals from single-cell data (see Appendix 5).

**Appendix 4—table 1.** Cell Cover marker genes extension at various depths.

1 H	Rbm20, Gm12481, Sox3, Tcf7l1, Rps18-ps3, Gm8129, Rps10-ps1, Rps13-ps2, Gm3362
24 H	Rnd2, Kit, Dusp14, Pramel7, Slc30a10, Sema6d, Sh3bgrl2, Neurod1, Nhlh1, Gm15232, Gm13789, Gm5619
96 H	Gabra1, Rasgrf2, Sla, Ntsr1, Clstn2, N28178, Fam135b, Fam19a2, Ccbe1, Adap1, Camk2b, mt-Tf, mt-Tv, mt-Nd4l, Gm15344, Nwd2, Gm26871
**(a) Covering markers of Telley dataset with d=3 and** ***α*****=0.02**	
1H	*Ajuba, Etl4, Rbm20, Gm12481, Sox3, Gas1, Rps18-ps3, Gm8129, Rps10-ps1, Rpl15-ps3, Rps13-ps2, Gm6654, Gm3362, Hspa1a*
24 H	*Rnd2, Kit, Dusp14, Serping1, Ifitm3, Pramel7, Slc30a10, Sema6d, Rasgef1b, Tmem176b, Crabp1, Neurod1, Mfap4, Sstr2, Nhlh1, 9630028B13Rik, Scrt2, mt-Ti, Adamtsl3, Gm12005, Gm15232, Gm13789, Gm5619*
96 H	*Mef2c, Gabra1, Tnr, Usp43, Rasgrf2, Sla, Dync1i1, Ptpro, Clstn2, N28178, Fam135b, Kcnj2, Fam19a2, Ccbe1, Adap1, Camk2b, Unc5c, mt-Tf, mt-Tv, mt-Nd4l, Gm15344, Nwd2, Gm26871, RP23-14P23.9*
**(b) Covering markers of Telley dataset with d=5 and** ***α*****=0.02**	
1 H	*Ajuba, Notch2, Rbm20, Gm12481, Sox3, Cenpe, Gas1, Tcf7l1, Hmga2, Rps18-ps3, Gm8129, Rps10-ps1, Rpl15-ps3, Sox21, Rps13-ps2, Gm6654, Gm3362, Hspa1a, WI1-1003N17.1*
24 H	*Rnd2, Kit, Dusp14, Gadd45g, Wnt7b, Serping1, Ifitm3, Pramel7, Tuba4a, Slc30a10, Sema6d, Rasgef1b, Cabp1, Tmem176b, Sh3bgrl2, Crabp1, Neurod1, Inf2, Ttc39b, Sstr2, Plcb1, Nhlh1, 9630028B13Rik, Scrt2, Adamtsl3, Gm12005, Rpl38-ps1, Gm15232, Gm13789, Pcp4, Gm5619*
96 H	*Mef2c, Gabra1, Tnr, Usp43, Rasgrf2, Sla, Dync1i1, Grip2, Ptpro, Clstn2, Arpp21, N28178, Fam135b, Dnm3, Rims1, Fam19a2, Ccbe1, Lingo1, Adap1, Camk2b, Unc5c, mt-Tf, mt-Tv, mt-Nd4l, Gm15344, Nwd2, Uba52, Gm26871, RP23-14P23.8*
**(c) Covering markers of Telley dataset with d=7 and** ***α*****=0.02**	
1 H	*Nr2e1, Zfp36l1, Spata13, Ajuba, Col2a1, Hes1, Flnb, Notch2, Cenpa, Gsta4, Aspm, Etl4, Rbm20, Gm12481, Sox3, Cenpe, Gas1, Cdca7, Dach1, Tcf7l1, Hmga2, Rps18-ps3, Gm8129, Rps26-ps1, Rps10-ps1, Rpl15-ps3, Sox21, Rpl34-ps1, Rps13-ps2, Sox2, Gm10224, Gm6654, Gm3362, Gm13267, Gm15795, Hspa1b, Hspa1a, Gm28625, WI1-1003N17.1*
24 H	*Rnd2, Kit, Dusp14, Nuak1, Gadd45g, Lrrc16b, Wnt7b, 5330426P16Rik, Serping1, Ifitm3, Pramel7, Tuba4a, Slc30a10, Sema6d, Necab3, Rasgef1b, Cabp1, Tmem176b, Sh3bgrl2, Crabp1, Eomes, Shf, Neurod1, Osbpl5, Inf2, Ttc39b, Clvs1, Sox5, Shb, Sstr2, Bcl11b, Nhlh2, Plcb1, Nhlh1, 9630028B13Rik, Rpl31-ps13, Cntn2, A930024E05Rik, Itpk1, Scrt2, Cxcl12, Unc5d, mt-Ti, Adamtsl3, Gm23935, Gm4294, Gm12005, Gm15481, Rpl38-ps1, Gm11989, Gm15232, Gm13789, Pcp4, Rps19-ps12, Gm5619*
96 H	*Ndrg1, Mef2c, Gabrb2, Crlf1, Met, Gabra1, Tnr, Glra2, Lama2, Gria1, Usp43, Rgs6, Mctp1, Rasgrf2, Cacna2d3, Sla, Cd200, Fbn2, Apba1, Prkar1b, Grin1, Dync1i1, Grip2, Grin2b, Ptpro, Slc6a11, Clstn2, Arpp21, Cck, Gucy1a3, N28178, Fam135b, Trim67, Dnm3, Cdh12, Necab1, Reps2, Rims1, Dlx1, 9330132A10Rik, Fam19a2, Ccbe1, Gpr85, Lingo1, Dlg2, Adap1, Fry, Ryr3, Camk2b, Unc5c, Kcnma1, mt-Tf, mt-Tv, mt-Nd4l, Nrxn3, A830039N20Rik, Scn2a1, Gm15344, Nwd2, Dlx6os1, Uba52, Gm26871, RP23-74F20.3, RP23-333L19.1, RP23-317L18.3, RP23-14P23.8, RP23-14P23.9*
**(d) Covering markers of Telley dataset with d=15 and α=0.02 extended from markers in Table Appendix 4-table1c**	
1 H	*Nr2e1, Zfp36l1, Gli3, Spata13, Ajuba, Col2a1, Hes1, Flnb, Notch2, Cenpa, Adamts9, Dusp16, Tead2, Sall1, Gsta4, Aspm, Etl4, Kif15, Ildr2, Rbm20, Gm12481, Sox3, Cenpe, Cldn12, Gas1, Creb5, Cdca7, Dach1, Tcf7l1, Hmga2, Gm7536, Rps18-ps3, Zfp516, Gm8129, Rps26-ps1, Rps10-ps1, Rpl15-ps3, Sox21, Phactr2, Rpl34-ps1, Rps13-ps2, Sox2, Gm10224, Gm6654, Gm3362, Gm13267, Gm15795, Hspa1b, Hspa1a, Gm26870, Gm28625, WI1-1003N17.1*
24 H	*Rnd2, Kcnn1, Kit, Syn2, Myt1, Dusp14, Nuak1, Gadd45g, Lrrc16b, Wnt7b, 5330426P16Rik, Serping1, Tmem178, Rcor2, Ifitm3, Nrp1, Pramel7, Tuba4a, Slc30a10, Sema6d, Necab3, Rasgef1b, Cabp1, Tmem176b, Nkd1, Sh3bgrl2, Crabp1, Eomes, Shf, Neurod1, Klhl35, Abca7, Ppp1r14a, Osbpl5, Inf2, Ttc39b, Clvs1, Sox5, Pxylp1, Shb, Rpsa-ps10, Sstr2, Bcl11b, Nhlh2, Plcb1, Nhlh1, 9630028B13Rik, Rpl31-ps13, Cntn2, A930024E05Rik, Itpk1, Scrt2, Cxcl12, Unc5d, mt-Ti, mt-Co3, Adamtsl3, Gm23935, Gm4294, Gm12005, Gm15481, Rpl38-ps1, Gm11989, Gm14094, Gm15232, Gm13789, Pcp4, Gm6467, Rps19-ps12, A730020E08Rik, Gm5619*
96 H	*Gabra2, Cacna1e, Chd5, Ndrg1, Mef2c, Gabrb2, Crlf1, Met, Gabra1, Tnr, Cacna1d, Glra2, Lama2, Gria1, Usp43, Rgs6, Mctp1, Rasgrf2, Cacna2d3, Sla, Cd200, Fbn2, Apba1, Prkar1b, Atp1b1, Grin1, Sparcl1, Dync1i1, Npy, Grip2, Grin2b, Ptpro, Slc6a11, Clstn2, Cspg5, Arpp21, Cck, Gucy1a3, Syt1, N28178, Fam135b, Trim67, Gabbr2, Dnm3, Cdh12, Necab1, Reps2, Inhba, 4930506M07Rik, Rims1, Dlx1, 9330132A10Rik, Fam19a2, Ccbe1, Gpr85, Lonrf2, Lingo1, Dlg2, Arhgap20, Adap1, Fry, Ryr3, Camk2b, Unc5c, Gm10115, Kcnma1, mt-Tf, mt-Tv, mt-Tl1, mt-Nd4l, Nrxn3, A830039N20Rik, 1810009A15Rik, Scn2a1, Gm15344, Gm15662, Nwd2, Dlx6os1, Uba52, Gm26871, RP23-74F20.3, RP23-333L19.1, RP23-317L18.3, RP23-14P23.8, RP23-14P23.9*
**(e) Covering markers of Telley dataset with d=20 and α=0.02 extended from markers in Table Appendix 4-table1d**	
1 H	*Otx1, Cdc20, Nr2e1, Plagl1, Grb10, Zfp36l1, Gli3, Samd4, Spata13, Ajuba, Col2a1, Hes1, Flnb, Notch1, Wwtr1, Notch2, Cenpa, Adamts9, Dusp16, Tead2, Sall1, Gsta4, Aspm, Etl4, Kif15, Mavs, Ildr2, Zic5, Dmrt3, Rbm20, Gm12481, Sox3, Cenpe, Cldn12, Gas1, Creb5, E330013P04Rik, Fgfr3, Cdca7, Dach1, Tcf7l1, Hmga2, Gm7536, Rps18-ps3, Zfp516, Gm8129, Rps26-ps1, Rps10-ps1, Rpl15-ps3, Sox21, Gm5453, Phactr2, Rpl34-ps1, Rps13-ps2, Sox2, Gm10224, Gm6654, Gm3362, Gm13267, Gm15795, Hspa1b, Hspa1a, Gm26870, 1190002F15Rik, Gm28625, WI1-1003N17.1*
24 H	*Sez6, Rnd2, Kcnn1, Kit, Syn2, Myt1, Dusp14, Nuak1, Chga, Gadd45g, Lrrc16b, Wnt7b, 5330426P16Rik, Serping1, Tmem178, Rcor2, Ifitm3, Nrp1, Pramel7, Tuba4a, Slc30a10, Sema6d, Necab3, Trp53inp1, Rasgef1b, Cabp1, Tmem176b, Nkd1, Sh3bgrl2, Crabp1, Eomes, Shf, Neurod1, Klhl35, Abca7, Ppp1r14a, Osbpl5, Inf2, Neurod6, Ttc39b, Neurod2, Nrn1, Ccser1, Clvs1, Sox5, Kif21b, Mfap4, Pxylp1, Shb, Rpsa-ps10, Sstr2, Bcl11b, Nhlh2, Plcb1, Nhlh1, 9630028B13Rik, Rpl31-ps13, Cntn2, A930024E05Rik, Itpk1, Rpl31-ps20, Scrt2, Cxcl12, Unc5d, mt-Ti, mt-Co3, Tecpr1, Hist2h2ac, Adamtsl3, Gm23935, Gm4294, Gm12005, Gm15481, Rpl38-ps1, Gm8606, Gm11989, Gm14094, Gm15232, Gm13789, Pcp4, Gm6467, Rps19-ps12, A730020E08Rik, Gm5619, 2600014E21Rik, Gm8885*
96 H	*Gabra2, Dlgap1, Cacna1e, Chd5, Ndrg1, Mef2c, Runx1t1, Gabrb2, Crlf1, Met, Gabra1, Prrt1, Tnr, Cacna1d, Glra2, Cacng2, Lama2, Gria1, Usp43, Rgs6, Mctp1, Rasgrf2, Cacna2d3, Sla, Cd200, Grm2, Nrxn1, Fbn2, Apba1, Prkar1b, Atp1b1, Grin1, Ntsr1, Ppp2r2c, Sparcl1, Dync1i1, Npy, Grip2, Grin2b, Ptpro, Tmtc1, Slc6a11, Clstn2, Cspg5, Arpp21, Cck, Gucy1a3, Lrfn5, Syt1, N28178, Fam135b, Trim67, March4, Gabbr2, Dnm3, Cdh12, Necab1, Reps2, Inhba, 4930506M07Rik, Rims1, Dlx1, Bend6, 9330132A10Rik, Fam19a2, Syt16, Amer3, Nxph1, Ccbe1, Gpr85, Lonrf2, Lingo1, Grm5, Dlg2, Arhgap20, Ppfia2, Cntn1, Maf, Adap1, Fry, Ryr3, Camk2b, Unc5c, Erbb4, Opcml, Gm10115, Kcnma1, mt-Tf, mt-Tv, mt-Tl1, mt-Nd4l, Nrxn3, Gad1, A830039N20Rik, 1810009A15Rik, Scn2a1, Gm15344, Gm15662, Nwd2, Dlx6os1, Uba52, A330076H08Rik, Gm26871, RP23-74F20.3, RP24-204F16.4, RP23-416H10.5, RP23-333L19.1, RP23-317L18.3, RP23-14P23.8, RP23-14P23.9*
**(f) Covering markers of Telley dataset with d=25 and α=0.02 extended from markers in Table Appendix 4-table1e**	

## Appendix 5

Currently, the vast majority of cell type markers derived from scRNA-seq data are selected from the top of a ranked list of genes differentially expressed between a cell class of interest versus other cell types (Dumitrascu et al., 2021; Hao et al., 2021). This approach has yielded many insightful results across tissue systems and species. While this method optimizes individual genes as cell type markers (by their DE rank) and creates a set of markers by selecting top individual genes that distinguish a cell type, CellCover optimizes a panel that functions together to define a cell class. This fundamental difference in the search for marker genes results in the capture of distinct cell-type specific signals. Therefore we put forth CellCover as a complementary approach to conventional DE marker gene finding, rather than a competing method for cell-type marker gene discovery.

Notable among the additional differences in these marker-finding methods is that CellCover (1) obviates the need for normalization, scaling or feature selection (as a function of utilizing binarized expression data), and (2) can directly capture heterogeneity within a cell class of interest. We show the latter empirically by comparing markers derived from CellCover and DE methods in the Telley data. Appendix 5—figure 1 summarizes the overlap of covering marker gene panels in Figure 2—figure supplements 1–4 (NeMO: Telley 12 CellCover Panels) and the Seurat DE ranked genes (NeMO: Telley 12 Sets DE Genes) for all 12 cell classes. In the majority of cell classes, there is appreciable divergence in the marker genes that the two methods identify as cell-type specific (represented by vertical gaps between the red and blue lines in each plot). Concordance between 96 H cell markers is greatest (perhaps indicating lower within-class heterogeneity in neurons), with the shortest list of top DE selected markers that include an entire covering marker gene panel at 40 genes in length (E13.96H). These observations indicate that in practice, the two methods of marker gene discovery produce quite divergent results.

To further explore the discordance in marker genes selected by the two methods, we visualized expression levels of individual E12.1H covering markers with high p-value rank in the DE (Appendix 5—figure 2 top panel) and those that are in the CellCover panel but rank lowly in the DE ranked list (Appendix 5—figure 2 bottom panel). We find markers with high DE rank, for example Hmga2 and Crabp2, are predominantly expressed in E12.1H cells, and they are also expressed in other cell populations, though less frequently. In contrast, genes appearing in the CellCover solution but at low rank in the DE are less frequently expressed but are more specific to the E12.1H class. For example, covering marker genes Nptx2, Pctp2, and Clnd9 are not as frequently expressed in E12.1H cells as other markers. However, these genes are rarely expressed in other cell classes. These marker genes with high specificity are very discriminative for the purpose of defining cell classes when used together but may be omitted by DE marker selection methods in the feature selection or DE calculation steps due to low and/or less frequent expression, i.e., low sensitivity as an individual marker. By optimizing the panel of marker genes rather than individual markers, CellCover can borrow power across multiple genes that show greater specificity but which individually do not identify all cells in a class.

To show this more systematically, we ran DE analysis on the 1 H, 24 H, and 96 H cells in the Telley data (Telley et al., 2019) and showed the rank of the CellCover markers (Figure 2A) based on the p-values returned from the DE analysis in Appendix 5—figures 3–5. We found that across all the cell ages, the CellCover markers that have low sensitivity also have a lower rank in the DE. For example, in the 1 H, the CellCover marker Hspa1a has the lowest sensitivity among all markers, expressed only near 13% of the time in this cell age, and its rank from DE is more than 500 (Appendix 5—table 1). Although this gene has low sensitivity, it has high specificity, rendering it a good candidate for a marker gene when used with other complementary genes. Genes with these properties appear at much lower ranks in DE and are hence not selected when using that method.

This ability to combine highly specific genes with individually low sensitivity – to generate marker panels of both high specificity and sensitivity – is responsible for much of the differences between gene panels selected by CellCover versus DE methods.

**Appendix 5—table 1.** Sensitivity analysis of CellCover 1 H markers. The first column of the table lists the average sensitivity of the marker gene in the cell population that is not 1 H, and the second column lists the sensitivity of the marker within class 1 H. The third column lists the p-value rank from DE. The CellCover marker with low sensitivity of 1 H also tends to have a low p-value rank in DE.

Gene	$ \frac{\sum_{l\neq k}P(U_{g}=1|Y=l)}{K-1}$	$P(U_{g}=1|Y=k)$	Rank in DE
Rps10-ps1	0.07	0.82	3
Gm3362	0.07	0.88	3
Gm6654	0.17	0.92	3
Rps18-ps3	0.10	0.88	3
Rps13-ps2	0.17	0.91	3
Gm8129	0.09	0.75	8
Sox3	0.10	0.70	17
Gas1	0.21	0.80	28
Gm12481	0.00	0.39	42
Etl4	0.15	0.67	57
Ajuba	0.08	0.49	108
Rpl15-ps3	0.03	0.37	121
Rbm20	0.05	0.40	149
Hspa1a	0.00	0.13	524

## Appendix 6

In addressing the challenge of marker gene selection, another approach, PhenotypeCover (Hasanaj et al., 2022), also draws inspiration from the set covering problem. While PhenotypeCover deploys multiset multicover to identify a global set of marker genes that discriminate all the cell types, CellCover captures cell-type specific signals whose combinatorial patterns characterize the gene expression behavior of the target cell sub-population, with the flexibility to extend to a global covering. A key distinction between the two methods lies in the resolution of the covering. CellCover defines covering at the level of both individual cells and cell-types, in contrast to the global cover of the entire cell population defined in the PhenotypeCover. This enables CellCover to better explore cellular heterogeneity and inspect the dynamics of different cell types in particular biological processes. Important mathematical distinctions between the two methods are also addressed in the following paragraphs.

The object to be covered are different in the two methods. CellCover defines covering at the level of both individual cell and cell-type. We say a cell is covered by marker panel $Q$ if more than $d$ genes in $Q$ are expressed in the cell. What is more, a cell type $k$ is covered if more than $1-\alpha$ proportion of the cells in this cell-type are covered. CellCover then identifies the optimal marker panel by minimizing the sum of weights of genes in the marker panel, where the weight of each gene reflects the inverse of its differentiating power of cell-type $k$, subject to the covering constraints on this cell-type. Therefore, CellCover identifies a marker panel for each cell-type such that the marker genes not only possess discriminative power of the phenotype but also cover the phenotype. Instead, PhenotypeCover identifies a global marker panel for the entire cell population such that the marker panel is of minimal cardinality and separates the phenotypes. In particular, in PhenotypeCover, a marker panel $Q$ provides a cover for the dataset if, for every phenotype pair $(i,j)$, the sum of weights of each gene in the marker panel exceeds $B$, where this weight of the gene depends on $(i,j)$ and is positively correlated with its discriminative power of this phenotype pair, and $B$ is a user-specified covering threshold. We note that CellCover also has a natural extension to identify a global marker set. To make a fair comparison, we will use the global version of the CellCover on the log normalized count data.

Let $N$ be the total number of cells and $G$ denote the set of genes. Given the gene expression matrix $X\in\mathbb{R}^{N\times|G|}$ and a known vector y of length N representing the phenotype. We derive $M_{i,g}$ from X by averaging the log normalized expression of gene g across all the cells with phenotype $i$. We also derive $U\in\{0,1\}^{N\times|G|}$ through binarization, where we let $u_{g}^{(c)}=1$ if $x_{g}^{(c)} > 0$ and 0 otherwise, and we say a gene is expressed if $u_{g}^{(c)}=1$. Further, we let $Z\in\{0,1\}^{|G|}$ to a binary vector so that $z_{g}=1$ means gene $g$ is selected in the marker panel. $Z$ is the variable we want to solve for in both methods. Next, we compare the mathematical forms of the two methods, and we will address the key differences from them.

### CellCover (global version)

In the CellCover, besides $Z$, we will also optimize another auxiliary binary variable $s^{c}$, where $s^{(c)}=1$ means that cell $c$ is covered. We also introduce two interpretable hyperparameters:

- $\boldsymbol{d}$: the covering depth. It is also the lower bound of the cardinality of the optimal covering panel.
- **α**: one minus the desired covering rate.

In the global version of CellCover, we define the weight to be $w_{g}=\min_{k}w_{g,k}$ where $w_{g,k}=\frac{\sum_{l\neq k}M_{g,l}/(K-1)}{M_{g,k}}$ and $M_{g,k}=\frac{\sum_{c\in S_{k}}x_{g}^{(c)}}{|S_{k}|}$. This weight reflects the maximum capacity of gene $g$ in differentiating one cell type versus the others. The integer program is given by

$$\displaystyle   \min_{Z}\sum_{g\in G}w_{g} z_{g} $$

$$\displaystyle   \sum_{g\in G}z_{g} u_{g}^{(c)}\geq \boldsymbol{d}s^{(c)}\quad c = 1,2,...N $$

$$\displaystyle   &\sum_{i=1}^{N} s^{(c)}\geq (1-\boldsymbol{\alpha})N $$

### PhenotypeCover

The hyperparameter in PhenotypeCover is $B$, a user-specified threshold of multiset multicover. The integer program is given by

$$\displaystyle   &\min_{Z}\sum_{g\in G}z_{g} $$

$$\displaystyle  \sum_{g\in G}|M_{g,i}- M_{g,j}|z_{g} \geq B \quad \forall \text{ cell-type pairs }(i,j) $$

Next, we list some key differences between the two methods:

- Weight scheme: The choices of weight are different from the two methods. CellCover uses the ratio of the average expressions to define the weight, whereas PhenotypeCover uses the absolute difference of the average expression as the weight. One immediate advantage of the CellCover weighting scheme is that it will encourage the algorithm to select genes that are specific to a phenotype which may have low overall expression rate. This may introduce some ‘noisy’ genes to the marker panel, but also help us to identify rare, lowly expressed genes that are equally important to characterize the cell-type. On the other hand, the Phenotype weighting scheme, together with its constraint, favors only those genes that are frequently expressed. The algorithm requires the sum of weights to exceed $B$ for all cell-type pairs. Including a lowly expressed but highly specific gene will not only make constraints in PhenotypeCover harder to satisfy but also incur a cost to the objective function. In contrast, CellCover encourages the identification of rare yet specific genes, and it also favors the genes that are frequently expressed and cell-type differentiating because the marker panel needs to satisfy the covering constraints. Hence, we should expect the covering to find a broader spectrum of marker genes regarding the rate of expression than the PhenotypeCover.
- Solver: CellCover solves the constraint optimization problem using integer programming (branch and bound algorithm), so the answer is (almost) exact when the primal objective and dual objective bounds converge. In particular, the returned solution is in some $\epsilon$ ball of the true optimal solution. PhenotypeCover solves the optimization by using the greedy approximation algorithm for multiset multicover, in which the accuracy of the solution is upper bounded by a factor of $H_{m}$ increase in the solution size where $H_{m}=1+1/2+...+1/m$ and $m$ are the cardinality of the largest multiset. CellCover provides a more accurate solution, but the branch and bound algorithm has a slower run time than the greedy algorithm used by PhenotypeCover, which is almost linear in the number of genes considered. However, we note that using the greedy algorithm to solve for the set covering problem inevitably undermines the combinatorial nature of the problem. When applying PhenotypeCover to real datasets, many returned solutions will fail to satisfy its constraints.
- The $B$ in the PhenotypeCover less directly informs the cardinality of the marker panel, and this threshold may differ from dataset to dataset due to differences in expression level. Determining a proper threshold in relation to the desired size of the marker panel requires trials and errors. In contrast, the hyperparameter $d$ in CellCover more directly informs the size of the marker panel, as it provides a lower bound to the number of markers in the optimal panel. Also, it is not dataset dependent.
- PhenotypeCover does not require covering on an individual cell level. Therefore, the method does not assess the interactions among the marker genes in each cell. CellCover imposes restrictions on the collective expression behavior of marker genes at the single-cell level. This constraint leverages the biological facts that co-expression of marker genes may activate certain functions.

We compare the two methods by applying them to obtain the global marker set of the Telley dataset. The goal is to obtain a set of marker genes for the entire dataset that distinguishes the time stamps 1 H, 24 H, and 96 H. For CellCover, we let $\boldsymbol{d}=20$ and $\boldsymbol{\alpha}=0.02$ to obtain a marker panel of size 61. For the PhenotypeCover, we set $B=95$ to get a marker set of the same size as CellCover. According to Appendix 6—figure 1, in comparison to PhenotypeCover which favors discriminative genes that are highly expressed, CellCover markers exhibit a larger range of probability of expression, encompassing not only highly expressed and discriminative genes but also encouraging the selection of rarely expressed yet highly specific genes.

## Appendix 7

The runtime performance of CellCover exhibits variability across different cell populations. This variability is not solely attributable to the size of the cell population under analysis. In practice, we have observed that CellCover can sometimes execute faster on datasets with larger cell populations than those with smaller ones. In general, the size of the effective search space of the candidate genes, the level of transcriptional distinctness of the cell type, and the size of the target cell population may jointly affect the runtime of our algorithm. Despite the potential for prolonged runtimes due to the NP-hardness nature of the branch and cut algorithm underlying the Gurobi solver, it is important to note that, once converged, CellCover guarantees a globally optimal solution. When the target cell population is large, even though we may not get a convergence certificate in a few seconds, the intermediate solution returned by Gurobi is guaranteed to be feasible (i.e., the corresponding gene panel will cover the target cell population) and can reach a stable suboptimal solution in a reasonable amount of time, typically within an hour in the worst case. A detailed analysis of the runtime is provided here, and we also outline a rule of thumb for determining the stopping criterion when the CellCover solver is slow on some cell populations.

- Size of the target cell population: The number of cells in a population of interest directly impacts the run time of covering. In CellCover, we define for each cell an auxiliary variable $s^{(c)}$ which indicates whether the cell $c$ is covered, and we will solve for this variable via integer programming. Hence, the number of variables $s^{(c)}$ scales with the size of the cell subpopulation, leading to a potential increase in runtime.
- Size of the effective search space of candidate genes: In the CellCover, we execute the pruning step before inputting the data into the Gurobi. Hence, the choice of filtering (see the Methods section) hyperparameters affects the size of the candidate genes, which directly impacts the run time of CellCover. First of all, we only select genes of which the fraction of cells expressing it is above $t_{e}$. Hence, a small $t_{e}$ may slow down the run time since the integer program needs to address the interplay of more genes. Secondly, for each cell type $k$, we select genes whose expression rates (the fraction of cells expressing the gene) within the subpopulation $k$ are higher than the average expression rates in other cell types. Equivalently, we filter out the genes with a margin less than the threshold $t_{d}$, for $t_{d}\geq 1$. In the implementation of the algorithm, since we might not know which $t_{d}$ is the desired cutoff, we execute this step of filtering by selecting the genes with the top $l$ margin in the target cell subpopulation that has a margin larger than 1. We observe that the requirement of $t_{d}\geq 1$ places the most significant filtering effect, sometimes reducing the number of candidate genes to the order of hundreds. As a result, the run time of CellCover scales with larger $l$ (or smaller $t_{d}$).
- The depth of covering $\boldsymbol{d}$: In general, a higher depth leads to an increase in run time because of the more difficult constraints to be satisfied. However, the impact of the covering depth also depends on the size of the candidate gene sets. If the covering depth is too high relative to the size of the effective search space of the genes, then the covering problem may become easier to solve, because the program will have to select a majority of the genes, if not all of them, to satisfy the constraints of covering. In this case, even though higher depth may surprisingly lead to a decrease in run time, the program becomes more susceptible to the feasibility problem because there may not be enough genes to cover the cell subpopulation at a large depth.

We augment this theoretical analysis with a run-time experiment on the Polioudakis dataset (Polioudakis et al., 2019) to extract CellCover markers from each of the 10 cell types. These experiments are run on a desktop with Intel 13,900 K CPU, and the results are shown in Appendix 7—figure 1.

Next, in case when the CellCover may take long to converge, we add features in the CellCover package which will guide the user to monitor the convergence of the program and implement stopping criteria to obtain stable, suboptimal solutions. When the CellCover starts running, it will output the log in real time. In the log, the metric ‘MIPGap’ will be the convergence indicator. By definition, it is the gap between the lower (dual) and upper objective bound (primal). The MIP solver will terminate with an optimal result when the gap between the MIPGap is sufficiently small. By default, the terminating MIPGap threshold is $10^{-4}$. To implement early stopping in the simplest way, we can either

- increase the gap threshold by setting the parameter argument ‘MIPGapThreshold’ in the python CellCover function to be any user-defined value.
- set a hard stopping time limit of the program by setting the parameter ‘TimeLimit’ (in s).

We note that every intermediate solution returned by the integer programming is guaranteed to be feasible, meaning that even the primal-dual gap on the objective is non-zero, the current gene panel solution ensures that the covering constraints are satisfied.

We also include additional features to the program that aid the users in monitoring the convergence and stableness of the solution in real time, and help the user to terminate the program with confidence. In particular, whenever the Gurobi improves its bound, we will periodically ask the program to print the current genes panel being found which is the best solution so far. Each time the program makes a callback, we also compute the size of the set difference between the current solution and the last solution. If the solution is converging, then this set difference will ideally become smaller. We let the program terminate if the set difference mentioned above is less than 3 for more than three consecutive callbacks. Users can also terminate the program themselves and obtain the most recent solution at their convenience.
