# Supplementary material for: CellCover defines marker gene panels capturing developmental progression in neocortical neural stem cell identity
Source: eLife. 2026 Jul 28;14:RP107531. doi: 10.7554/eLife.107531 (PMC13412324; doi:10.7554/eLife.107531)
Supplement: Peer review history

# Editor report: eLife Assessment

**DOI:** 10.7554/eLife.107531.2.sa0

**Authors:** Murim Choi

**Affiliations:** Seoul National University Republic of Korea

**Keywords:** Compelling, Valuable

This study offers a **valuable** methodological advance by introducing a gene panel selection approach that captures combinatorial specificity to define cell identity. The findings address key limitations of current single-gene marker methods. The evidence is **compelling**, but would be strengthened by further validation of rare cell states and unexpected marker categories.

# Referee report: Joint Public Review

**DOI:** 10.7554/eLife.107531.2.sa1

In this study, the authors introduce CellCover, a gene panel selection algorithm that leverages a minimal covering approach to identify compact sets of genes with high combinatorial specificity for defining cell identities and states. This framework addresses a key limitation in existing marker selection strategies, which often emphasize individually strong markers while neglecting the informative power of gene combinations. The authors demonstrate the utility of CellCover through benchmarking analyses and biological applications, particularly in uncovering previously unresolved cell states and lineage transitions during neocorticogenesis.

The major strengths of the work include the conceptual shift toward combinatorial marker selection, a clear mathematical formulation of the minimal covering strategy, and biologically relevant applications that underscore the method's power to resolve subtle cell-type differences. The authors' analysis of the Telley et al. dataset highlights intriguing cases of ribosomal, mitochondrial, and tRNA gene usage in specific cortical cell types, suggesting previously underappreciated molecular signatures in neurodevelopment. Additionally, the observation that outer radial glia markers emerge prior to gliogenic progenitors in primates offers novel insights into the temporal dynamics of cortical lineage specification.

However, several aspects of the study would benefit from further elaboration. First, the interpretability of gene panels containing individually lowly expressed genes but high combinatorial specificity could be improved by providing clearer guidelines or illustrative examples. Second, the utility of CellCover in identifying rare or transient cell states should be more thoroughly quantified, especially under noisy conditions typical of single-cell datasets. Third, while the findings on unexpected gene categories are provocative, they require further validation - either through independent transcriptomic datasets or orthogonal methods such as immunostaining or single-molecule FISH-to confirm their cell-type-specific expression patterns.

Specifically, the manuscript would benefit from further clarification and additional validation in the following areas:

• A more in-depth explanation of marker panel applications is needed. Specifically, how should users interpret gene panels where individual genes show only moderate or low expression levels, but the combination provides high specificity? Providing a concrete example, along with guidelines for interpreting such combinatorial signatures, would enhance the practical utility of the method.

• Further quantification of CellCover's sensitivity in detecting rare cell subtypes or states would strengthen the evaluation of its performance. Additionally, it would be helpful to assess how CellCover performs under noisy conditions, such as low cell numbers or read depths, which are common challenges in scRNA-seq datasets.

• It is intriguing and novel that CellCover analysis of the dataset from Telley et al. suggests cell-type-specific expression of ribosomal, mitochondrial, or tRNA genes. These findings would be significantly strengthened by additional validation. For example, the reported radial glia-specific expression of Rps18-ps3 and Rps10-ps1, as well as the postmitotic neuron-specific expression of mt-Tv and mt-Nd4l, should be corroborated using independent scRNA-seq or spatial transcriptomic datasets of the developing neocortex. Alternatively, these expression patterns could be directly examined through immunostaining or single-molecule FISH analysis.

• The observation that outer radial glia (oRG) markers are expressed in neural progenitors before the emergence of gliogenic progenitors in primates and humans is compelling. This could be further supported by examining the temporal and spatial expression patterns of early oRG-specific markers versus gliogenic progenitor markers in recent human spatial transcriptomic datasets - such as the one published by Xuyu et al. (PMID: 40369074) or Wang et al. (PMID: 39779846).

Summary:

Overall, this work provides a conceptually innovative and practically useful method for cell type classification that will be valuable to the single-cell and developmental biology communities. Its impact will likely grow as more researchers seek scalable, interpretable, and biologically informed gene panels for multimodal assays, diagnostics, and perturbation studies.

# Author comment: Author response

**DOI:** 10.7554/eLife.107531.2.sa2

**Authors:** Lanlan Ji, An Wang, Shreyash Sonthalia, Seungmae Seo, Daniel Q Naiman, Laurent Younes, Carlo Colantuoni, Donald Geman

**Affiliations:** Johns Hopkins University Baltimore United States; Johns Hopkins University Baltimore United States; Johns Hopkins University Baltimore United States; University of Maryland Eastern Shore Princess Anne United States; Johns Hopkins University Baltimore United States; Johns Hopkins University Baltimore United States; Johns Hopkins University BALTIMORE United States; Johns Hopkins University Baltimore United States

A more in-depth explanation of marker panel applications is needed. Specifically, how should users interpret gene panels where individual genes show only moderate or low expression levels, but the combination provides high specificity? Providing a concrete example, along with guidelines for interpreting such combinatorial signatures, would enhance the practical utility of the method.

We appreciate the need to explain and demonstrate how to use the novel combinatorial gene marker sets that CellCover generates. To be clear, individual genes expressed at low levels and in small numbers of cells, in general, have high specificity (the ability to mark cells of a particular type without erroneously marking other cells as this type) and are often used in combinations by CellCover to achieve a panel of genes with high sensitivity (the ability to mark all cells of a particular type). Low or sparsely expressed genes of this type may represent poorly measured genes (i.e. zero inflation known to occur in single-cell data, where genes are measured as zero in cells which actually express the gene) or may represent genes which are truly expressed only in a subset of the annotated class. Because CellCover can borrow strength across genes, it can harness the true information in either class of genes, even if affected by zero inflation. Further investigation of structure within the cell class (and across other cell classes) using the CellCover gene marker panel, as well as other genes, is necessary to clarify this issue in any particular analysis. In the manuscript, we evaluate the expression of individual genes within and across classes in this manner to understand deeper structure in Figures 1A, S6 and S8.

To demonstrate how CellCover selects individual genes with high specificity and low sensitivity, but which are complementary to one another, in order to achieve high collective sensitivity, here we consider a hypothetical dataset of many cells where we focus on one cell class that contains 100 cells composed of four subtypes.

- Subtype A: cells 1–20

- Subtype B: cells 21–30

- Subtype C: cells 31–50

- Subtype D: cells 51–100

To illustrate how CellCover evaluates marker gene panels, in this example, the genes under instigation have very different weights (i.e. the ratio of a gene’s expression in the cell class of interest versus its expression in other cells). Suppose we have two candidate marker panels:

Panel 1 (coarse markers).

- Gene *A*: covers cells 1–30 (weight = 0.4)

- Gene *B*: covers cells 30–60 (weight = 0.3)

- Gene *C*: covers cells 60–100 (weight = 0.2)

Each gene in this panel covers a relatively large portion of the population (*>* 30%), but their weights are comparatively high, indicating limited specificity to the focal cell type. Although the panel {*A,B,C*} attains full coverage, its markers are coarse and nonspecific.

Panel 2 (fine-grained, combinatorial markers).

- Gene *A*’: covers cells 1–20 (weight = 0.05)

- Gene *B*’: covers cells 20–30 (weight = 0.10)

- Gene *C*’: covers cells 30–50 (weight = 0.05)

- Gene *D*’: covers cells 50–100 (weight = 0.10)

Each marker is expressed in a smaller fraction of the population (individually low sensitivity), but the weights are substantially lower, reflecting strong subtype specificity. Importantly, these genes are complementary: their union covers all 100 cells (high combinatorial sensitivity), even though no single gene spans more than 20–50% of the cells.

Under a strict covering requirement (e.g., *α* = 0, requiring 100% coverage, i.e. perfect sensitiity), both panels satisfy the constraint. However, CellCover selects the second panel because its total weight (specificity) is smaller. This preference reflects the design of the objective function: the method favors markers that are highly cell-type-specific, even if they individually cover only a subset of the population, as long as their complements yield full coverage. As a result, CellCover can reveal refined subtype structure within what appears to be a single cell population.

Interpretation guidelines. We explicitly note that CellCover marker panels should be interpreted as combinatorial signatures:

- Individual genes may show localized, subtype-restricted expression.

- The union of their expression defines the target cell type.

- Low-weight genes are more specific; CellCover therefore prioritizes them whenever they provide complementary coverage.

- The resulting panel may highlight latent heterogeneity or subpopulations within the cell type that express different subsets of the markers.

In addition to these technical guidelines for interpreting gene panels, throughout the manuscript we use the transfer of CellCover marker gene panels to related datasets to assess the biological function of the gene sets. We propose this as a general tool in the examination of gene lists and have implemented methods to visualize the expression of any gene list (including gene lists uploaded by users) using the Projection Tool within NeMO Anlaytics.

Further quantification of CellCover’s sensitivity in detecting rare cell subtypes or states would strengthen the evaluation of its performance. Additionally, it would be helpful to assess how CellCover performs under noisy conditions, such as low cell numbers or read depths, which are common challenges in scRNA-seq datasets.

While CellCover is a method to define marker gene panels for cell classes that are already defined in a dataset, its performance on rare cell classes, small numbers of cells and low read depths is still a relevant issue. The analyses in the paper can speak to some of these concerns: The Telley dataset, which we use throughout the manuscript, used FlashTag labeling of cells prior to sequencing in order to ascertain the time since terminal division for each cell. This unique metadata linked to each cell’s expression data enabled many of the analyses we performed in the paper, but also limited the number of cells that were sequenced. For this reason, the number of cells in this dataset (total cells = 2756) is much lower than that seen in the vast majority of other single-cell sequencing studies, including those we use for the transfer of marker gene sets defined by CellCover in the Telley data. As a result, the cell classes for which we define marker gene panels in the paper contain relatively small numbers of cells. This is especially true in the 12-class analysis in Figures 4 and 5 where CellCover successfully defines gene panels for all 12 classes which transfer well to other datasets. Total cells per class range from 134 to 301. Figure S6 shows that the discriminative power of the 12 gene panels varied widely, with the most highly discriminative panel being from the E12.1H condition with only 189 cells.

In addition, we note that the behavior of CellCover on rare (or any) cell classes can be characterized deterministically under mild condition. For a fixed cell class and a required covering rate of 1, a depth-k covering gene panel exists if and only if every cell in the class expresses at least k genes. Under this condition, CellCover is guaranteed to find a covering panel of depth-k. Importantly, this guarantee does not impose any restriction on the panel size. Consequently, the compactness of the resulting panel reflects intrinsic properties of the data rather than algorithmic limitations: a small panel indicates that a subset of genes is robustly and consistently expressed across most cells in the class, even if the class itself is rare, whereas a large panel suggests highly heterogeneous expression patterns, where different genes are expressed in different cells. In this sense, the feasibility and structure of a covering panel are determined by the biological and technical characteristics of the dataset (e.g., read depth, expression sparsity, and the specificty of gene expression in the defined cell classes), rather than by the performance of CellCover itself.

It is intriguing and novel that CellCover analysis of the dataset from Telley et al. suggests cell-type-specific expression of ribosomal, mitochondrial, or tRNA genes. These findings would be significantly strengthened by additional validation. For example, the reported radial glia-specific expression of Rps18-ps3 and Rps10-ps1, as well as the postmitotic neuron-specific expression of mt-Tv and mt-Nd4l, should be corroborated using independent scRNA-seq or spatial transcriptomic datasets of the developing neocortex. Alternatively, these expression patterns could be directly examined through immunostaining or single-molecule FISH analysis.

The main problem with such analysis is that most studies have omitted the expression of these genes (especially mitochondrial genes that are primarily viewed as QC metrics) from their datasets. We encourage researchers to retain the expression of these transcripts in their data so that their biological functions can be explored. Where available, the expression of these genes can be visualized in NeMO Analytics in the mouse where the enrichment of Rps18-ps3 expression in radial glia can be seen in the Di Bella 2021 dataset and in the human where the expression of mt-Tv can be seen in neurons in the Polioudakis 2019, Darmanis 2015, Camp 2015, and Liu 2016 datasets.

Taking a broader perspective, a growing body of foundational work in developmental neurobiology supports the observation that mitochondrial state and metabolic programs undergo systematic changes during neuronal differentiation, consistent with our CellCover findings. For example, Khacho 2016 demonstrated that mitochondrial dynamics are essential regulators of neuronal fate commitment and that the maturation of the mitochondrial network is essential for the transition from the progenitor metabolic state to the neuronal state. Iwata 2020 further highlight cell type specific mitochondrial dynamics by showing that daughter cells with highly fragmented mitochondria tend to become neurons.

The observation that outer radial glia (oRG) markers are expressed in neural progenitors before the emergence of gliogenic progenitors in primates and humans is compelling. This could be further supported by examining the temporal and spatial expression patterns of early oRG-specific markers versus gliogenic progenitor markers in recent human spatial transcriptomic datasets - such as the one published by Xuyu et al. (PMID: 40369074) or Wang et al. (PMID: 39779846).

We have added the scRNA-seq data from Wang et al., as well as data from the Nano et al. 2025 meta-atlas to the NeMO Analytics data collection. oRG markers from Liu et al 2023 can now be visualized across the Wang, Nano and many more human in vivo datasets. In the Nano data, these oRG markers can be seen increasing in expression in the human neocortex from GW7-12, leading into peak neurogenesis and prior to gliogenesis. Although with lower age resolution, the peaking of oRG markers in the 2nd trimester (dring peak neurogenesis) and their precipitous drop in the 3rd trimester (during peak gliogenesis) can also be seen in the Wang data. At NeMO Analytics individual marker genes of oRGs can also visualized in these datasets.
